# Circular RNA circMagi1 regulates the host immune response in respiratory *Pseudomonas aeruginosa* infection through G3BP2

**DOI:** 10.1128/mbio.03617-25

**Published:** 2026-03-23

**Authors:** Miao Tang, Yongxin Zhang, Rou Xie, Tong Wu, Peng Sun, Yongxiao Luo, Ruihuan Wang, Teng Ma, Yang Yasmine Yang, Yige Zhang, Chaoyu Zou, Xueli Hu, Jing Shirley Li, Huan Liu, Zhuochong Liu, Qianhua Zhang, Yu Tang, Hongliang Li, Yilin Liu, Yang Yang, Jing Li, Ce Bian, Yongjiang Tang, Xikun Zhou

**Affiliations:** 1 Department of Biotherapy, Cancer Center and State Key Laboratory of Biotherapy, West China Hospital, Sichuan University 12530, Chengdu, China; 2 Department of Pulmonary and Critical Care Medicine, West China Hospital, Sichuan University 12530, Chengdu, China; 3 Department of Ophthalmology, West China Hospital, Sichuan University 12530, Chengdu, China; 4 State Key Laboratory of Oral Diseases, National Clinical Research Center for Oral Diseases, Chinese Academy of Medical Sciences Research Unit of Oral Carcinogenesis and Management, West China Hospital of Stomatology, Sichuan University 12530, Chengdu, China; 5 Key Laboratory of Birth Defects and Related Diseases of Women and Children, Department of Gynecology and Obstetrics, Ministry of Education, West China Second Hospital, Sichuan University 12530, Chengdu, China; 6 Laboratory of Pathogen Research, West China Hospital, Sichuan University 12530, Chengdu, China; 7 Frontiers Medical Center, Tianfu Jincheng Laboratory 661035 , Chengdu, China; Cornell University, Ithaca, New York, USA

**Keywords:** *Pseudomonas aeruginosa*, infection immunity, circMagi1, G3BP2

## Abstract

*Pseudomonas aeruginosa* is a major pathogen responsible for hospital-acquired infections, and it is characterized by possessing easy colonization ability, being difficult to clear, and exhibiting pandrug resistance. After a host is infected with pathogenic bacteria, multiple molecules regulate immune defense mechanisms through synergistic or antagonistic effects. Circular RNAs (circRNAs), characterized by their closed loop structure, are noncoding RNAs that are extensively expressed in various tissues. They have been reported to play a role in a range of physiological and pathological processes within the host. However, their involvement in regulating the immune activation mechanism against bacterial infection remains uncertain. In this study, we identified circMagi1, a circRNA that is significantly downregulated after infection in both a mouse model of lung infection and patients with lung infections. circMagi1 functions mainly as a negative regulator of immune activation for immune homeostasis. Further research has shown that it affects the cytokine signaling axis in macrophages by regulating the G3BP2 protein. Therefore, circMagi1 may function as a promising diagnostic biomarker for bacterial pathogen infections.

**IMPORTANCE:**

Circular RNAs (circRNAs) have been reported to be widely involved in the occurrence and development of various diseases, including cancer and neurodegenerative diseases, but there are few reports on circRNAs regulating host immune activation in bacterial infection. On the other hand, in contrast to the well-characterized progress and treatment of the disease and the prevalence of bacterial resistance in *Pseudomonas aeruginosa* pulmonary infection in recent decades, research on the mechanism of host-pathogen interactions and the regulatory mechanism of the host immune microenvironment has gained less attention. In this study, we constructed a mouse model of *P. aeruginosa* lung infection and analyzed patient alveolar lavage fluid samples, identifying a circRNA that is significantly downregulated postinfection. Our further investigation revealed that circMagi1 interacts with the G3BP2 protein, increasing its stability by inhibiting its ubiquitination. This newly discovered mechanism helps us understand how the host prevents excessive immune activation during bacterial infection.

## INTRODUCTION

Bacterial infections are a compilation of various diseases and are becoming the second leading cause of death globally, following ischemic heart disease. In 2019, more than 50% of global deaths from bacterial infections were caused by a few key pathogens, including *Staphylococcus aureus*, *Escherichia coli*, *Streptococcus pneumoniae*, *Klebsiella pneumoniae*, and *Pseudomonas aeruginosa* (1). Among them, *P. aeruginosa* is the most common pathogen causing clinical respiratory infections (2, 3). Compared with other bacteria, *P. aeruginosa* possesses a larger genome and can acquire numerous genes through horizontal gene transfer. Its extensive and complex regulatory mechanisms enable high adaptability to environmental changes, contributing to the emergence of multidrug-resistant strains (4–8). Currently, there are no effective treatments or drugs specifically targeting *P. aeruginosa* (9). Infections caused by this pathogen often result in immune dysfunction, including immune hyperactivity and immune suppression (10), although the precise underlying mechanisms remain poorly understood. After infection, various molecules regulate the immune defense process through synergistic or antagonistic mechanisms. However, the identification of relevant regulatory molecules that govern the host’s immune response to bacterial infections remains incomplete. Therefore, a thorough exploration of the regulatory mechanisms involved in host immune defense is crucial for the control of pathogen infection.

Circular RNAs (circRNAs) are a type of endogenous noncoding RNA characterized by a covalently closed loop structure lacking a 5′ cap and 3′ poly(A) tail (11, 12). Most circRNAs are derived from known genes and can be categorized into three types: exonic, intronic, and exon-intron circRNAs (13). Compared to linear RNAs, circRNAs are more stable, exhibit longer half-lives, and display widespread, abundant, spatiotemporally specific expression. These unique characteristics enable circRNAs to play critical and irreplaceable roles in biological processes (14, 15). circRNAs have been implicated in the progression of various pathological conditions, including autoimmune diseases, cardiovascular diseases, nervous system diseases, and cancers (16). Moreover, some circRNAs have potential as diagnostic tools or targets for specific therapies (17–19). However, the role of circRNAs in regulating host immune defense against bacterial infection remains poorly understood.

Magi1 (membrane-associated guanylate kinase with WW and PDZ domain-containing protein 1) was initially reported as an intracellular scaffolding protein involved in stabilizing epithelial junctions and later found to be widely involved in the physiological and pathological processes of tumor suppression (20), neural development (21), and immune response regulation (22). In this study, we discovered that human and mouse Magi1 genes can encode highly homologous circRNAs, circMagi1 (human hsa_circ_0066459 and mouse mmu_circ_0001496). We further revealed that circMagi1 participates in the host immune response by regulating macrophage inflammatory factor expression through G3BP2 and affecting the activation of immune cells in lung tissue during *P. aeruginosa* infection.

## RESULTS

### Identification and characteristics of circMagi1 during *P. aeruginosa* infection

**Fig 1:**
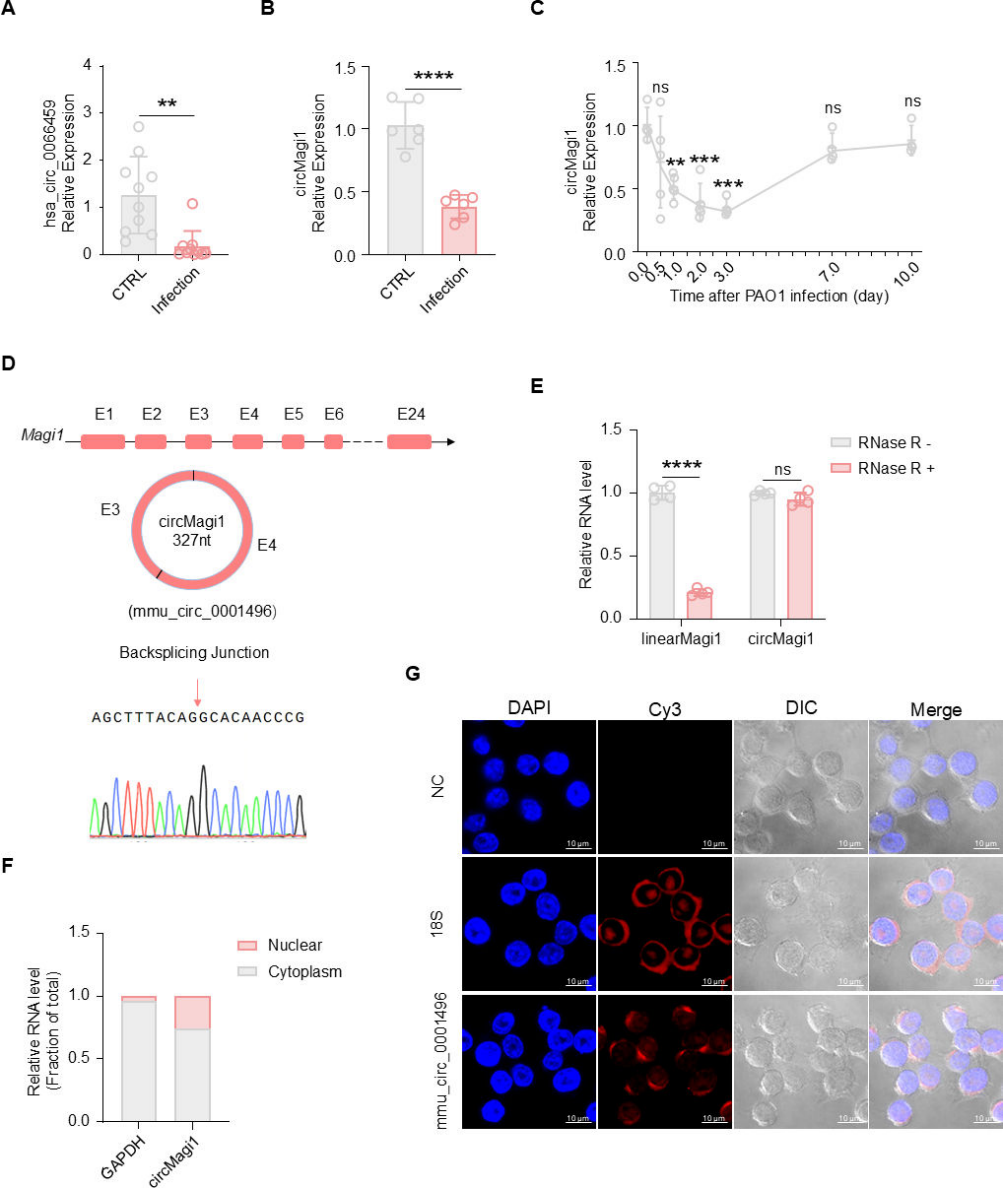
Identification and characteristics of circMagi1. (**A**) qPCR detection of circMagi1 (hsa_circ_0066459) levels in patient alveolar lavage fluid. Patient samples were divided into CTRL group (*n* = 10) and Infected group (*n* = 10) on the basis of the presence or absence of *P. aeruginosa* lung infection, with each point representing one sample. (**B**) qPCR detection of circMagi1 levels in mouse lungs. The infection and CTRL groups each had six mice; each mouse was subjected to a *P. aeruginosa* lung infection via oropharyngeal aspiration of 2 × 10^6^ CFU, and whole lungs were collected for analysis 24 h after infection. (**C**) qPCR detection of circMagi1 levels in mouse lungs at different infection time points after *P. aeruginosa* lung infection. There were four to five mice at each time point, and each mouse was infected with 3 × 10^6^ CFU. (**D**) Schematic diagram of the structure of circMagi1. (**E**) qPCR detection of the stability of circMagi1 and linearMagi1 after RNase treatment. (**F**) qPCR detection of the cytoplasmic and nuclear localization of circMagi1. (**G**) FISH detection of the cytoplasmic and nuclear localization of circMagi1 using Cy3-labeled circMagi1 and 18S rRNA probes. All data are presented as the mean ± SD. Statistical significance was assessed using unpaired *t* tests with Welch’s correction if necessary (**A, B, E**) or one-way ANOVA (**C**) with comparison to the 0 h control. NC, negative control; ns, not significant; ****P < 0.0001, ***P < 0.001, **P < 0.01, and *P < 0.05.

In patients with bacterial lung infection, the expression of circMagi (hsa_circ_0066459) was significantly reduced in bronchoalveolar lavage fluid (BALF) samples compared to uninfected controls (Table S2; Fig. 1A). Given the stable structure and high evolutionary conservation of circRNAs (23), we compared the sequence of circMagi1 (mmu_circ_0001496) in mice with its human homolog (hsa_circ_0066459) and found a high sequence similarity of 94.26% (Fig. S1). This conservation suggested that *P. aeruginosa* infection might similarly downregulate mmu_circ_0001496 in mice. To test this, we established a *P. aeruginosa* lung infection model in mice via oropharyngeal aspiration of 2 × 10^6^ CFU PAO1 per mouse and observed a significant reduction in circMagi1 (mmu_circ_0001496) expression in lung tissues 24 h post-infection (Fig. 1B). To further investigate the expression dynamics of circMagi1 following infection, lung tissues were collected at multiple time points. Total RNA was extracted, and circMagi1 levels were measured using specific primers. The results showed a gradual decrease in circMagi1 expression during the early stages of infection, followed by a return to baseline levels during the recovery phase (Fig. 1C). These findings suggest a potential relationship between circMagi1 expression and the immune activation during bacterial infection to some extent (24, 25) and indicate a potentially conserved role for circMagi1 in immune regulation across both humans and mice (13).

Backsplicing is the primary mechanism for circRNA biogenesis (12). circMagi1 is a closed-loop structure of 327 nt that is formed by backsplicing of exons E3 and E4 of the Magi1 gene (Fig. 1D). circMagi1 exhibited significant resistance to RNase R treatment, demonstrating greater stability than its linear transcript (Fig. 1E). Subcellular fractionation followed by qPCR further revealed that circMagi1 was approximately three times more abundant in the cytoplasm than in the nucleus in immortalized bone marrow-derived macrophages (iBMDMs).

As a reference, approximately 95% of the GAPDH transcript was detected in the cytoplasm and 5% in the nucleus (Fig. 1F). Moreover, RNA fluorescence *in situ* hybridization (FISH) further confirmed that circMagi1 is expressed in both the cytoplasm and nucleus, with primary localization in the cytoplasm (Fig. 1G).

### Overexpression of circMagi1 promotes the expression of macrophage inflammatory factors

**Fig 2:**
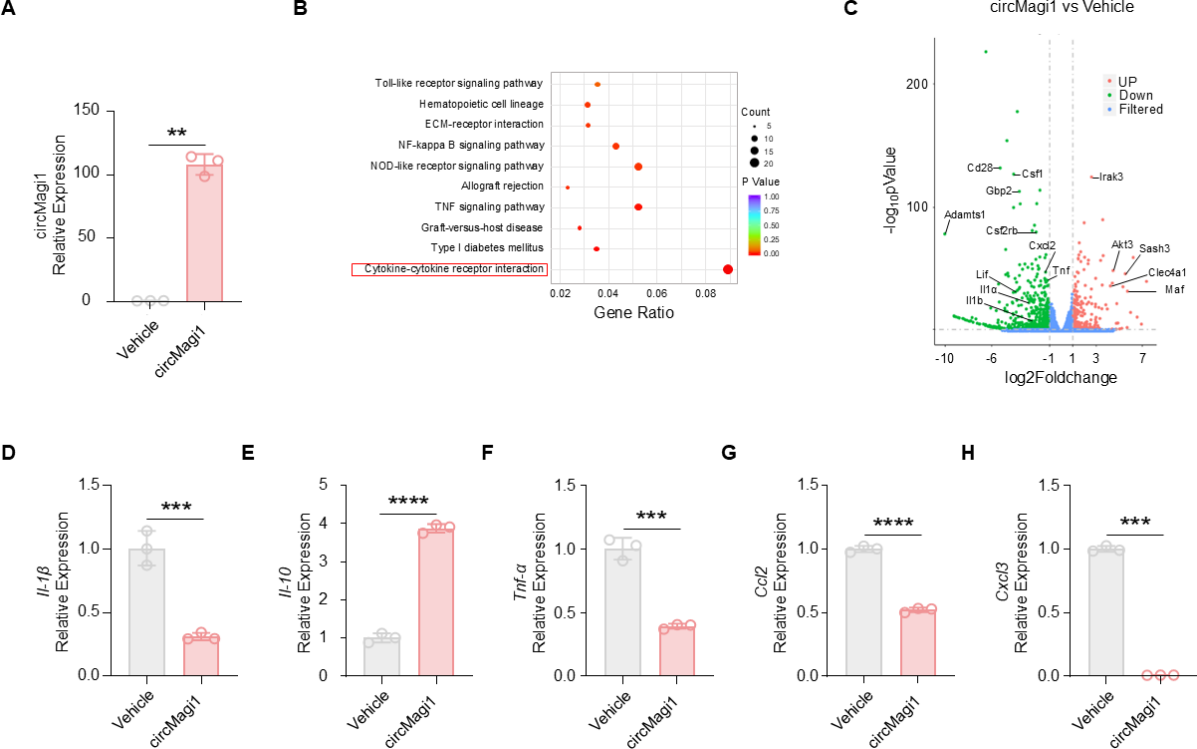
Overexpression of circMagi1 regulates the expression of macrophage inflammatory factors. (**A**) qPCR detection of circMagi1 after circMagi1 was overexpressed in iBMDMs. (**B**) Bubble plot of gene enrichment analysis for transcriptome sequencing of pLVX and pLVX-circMagi1 iBMDMs. Each group had three replicates, and all the samples were treated with PAO1 (MOI = 20) for 6 h before collection. (**C**) Volcano plot of the transcriptome sequencing data. (**D–H**) qPCR detection of Il-1β, Il-10, Tnf-α, Ccl2, and Cxcl3 after circMagi1 was overexpressed in iBMDMs. The cells were treated with PAO1 for 6 h before detection. All data are presented as the mean ± SD. Statistical significance was assessed using unpaired *t* tests with Welch’s correction if necessary (**A, D–H**). ****P < 0.0001, ***P < 0.001, and **P < 0.01. iBMDM, immortalized bone marrow-derived macrophage.

Macrophages are main immune cells involved in the host immune response to pathogenic bacterial infection and play a crucial role in maintaining immune homeostasis (26–29). To further investigate the functional role of circMagi1 in macrophages, we constructed a pLVX vector to overexpress circMagi1 and used lentiviral packaging to infect iBMDMs. Following puromycin selection, we established an iBMDM cell line with circMagi1 expression levels approximately 100-fold higher than those in the control group (Fig. 2A). After treatment with PAO1, the cells were collected for transcriptome sequencing, and KEGG enrichment analysis was performed on the differentially expressed genes (DEGs). The analysis revealed that the cytokine‒cytokine receptor interaction pathway is the most significantly enriched pathway between the two groups (Fig. 2B; Table S3). Compared to the control group, the circMagi1-overexpressing group exhibited lower levels of multiple proinflammatory cytokines, including Il-1α, Tnf, and Cxcl2 (Fig. 2C). Further qPCR analysis of PAO1-infected cells confirmed significantly reduced expression of proinflammatory cytokines, such as Il-1β, Tnf-α, Ccl2, and Cxcl3 in circMagi1-overexpressing cells compared to control cells. In contrast, the expression of the anti-inflammatory cytokine Il-10 was significantly upregulated (Fig. 2D through H). These findings suggest that circMagi1 suppresses proinflammatory cytokine expression in macrophages, indicating that it may act as an anti-inflammatory molecule to mitigate inflammatory responses.

### circMagi1 affects host immune function *in vivo*

**Fig 3:**
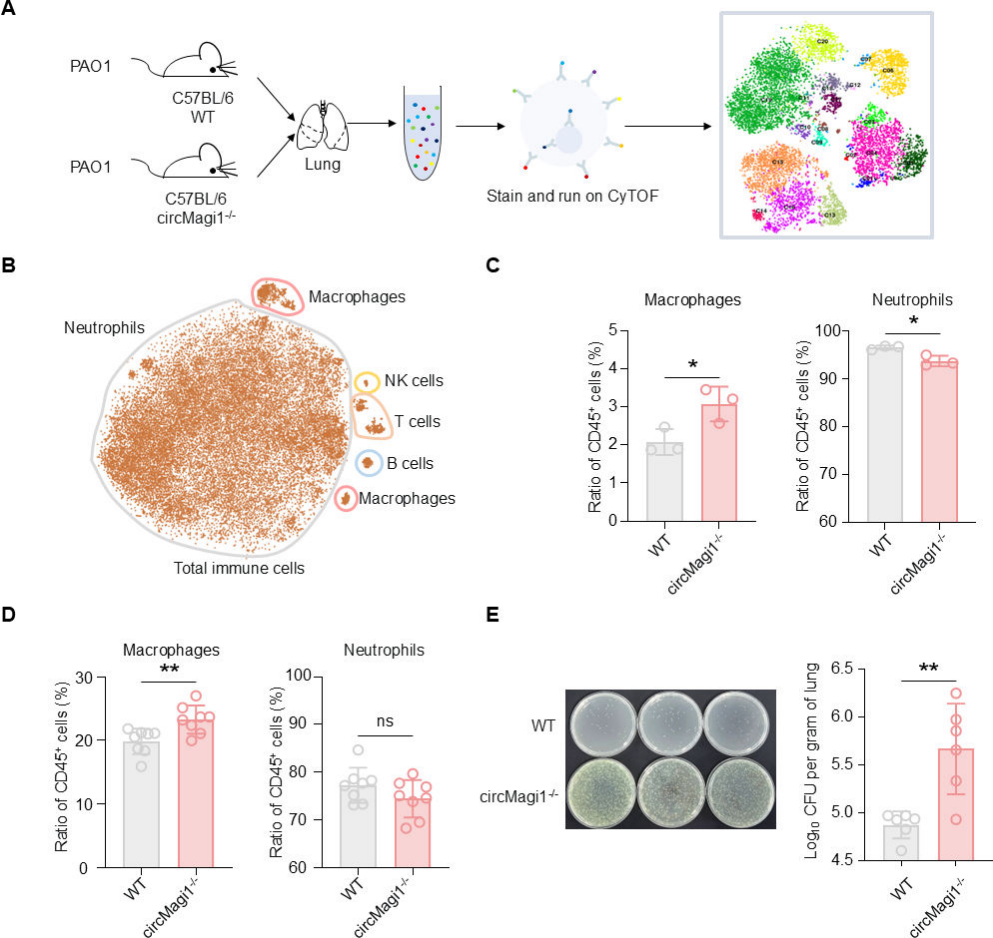
Knockdown of circMagi1 affects the host immune response to bacterial infection *in vivo*. (**A**) Schematic diagram of CyTOF immunoprofiling analysis of mouse lung tissues. (**B**) Scatterplot of two-dimensional t-SNE visualization of immune cells in mouse lung tissues. (**C**) CyTOF analysis of macrophages and neutrophils within the CD45^+^ population. Each group included three mice, each mouse was infected with 4 × 10^6^ CFU, and whole lungs were collected for detection 24 h after infection. (**D**) Flow cytometry analysis of macrophage and neutrophil proportions in the wild-type (WT) mouse and the circMagi1^−/−^ mouse, with eight mice in each group. Each mouse was infected with 3 × 10^6^ CFU, and whole lungs were collected for detection 24 h after infection. (**E**) Bacterial load in lungs of wild-type and circMagi1^−/−^ mice. Mice (*n* = 6 per group) were infected with 3 × 10^6^ CFU PAO1 and lungs were harvested 24 h post-infection for CFU analysis. Left: quantitative CFU counts. Right: representative bacterial culture plates from WT and circMagi1^−/−^ mice. All data are presented as the mean ± SD. Statistical significance was assessed using unpaired *t* tests with Welch’s correction if necessary (**C–E**). ns, not significant; ****P < 0.0001, **P < 0.01, and *P < 0.05. CyTOF, cytometry by time-of-flight.

To further explore the role of circMagi1 in host immunity, we constructed circMagi1 knockout mice via CRISPR-Cas9 technology. By deleting a specific intronic sequence of the *Magi1* gene in C57BL/6 mice, we disrupted circMagi1 production by removing the intronic sequence necessary for its circularization (Fig. S2A and B). Meanwhile, we retained the exon sequences required for the normal splicing of the linear Magi1 transcript, ensuring that its expression remains unaffected (Fig. S2C and D). After genotyping the knockout mice, homozygous mice were bred for subsequent experiments. Lung tissue analysis showed that the expression of cytokines such as Il-1β and Tnf-α was significantly increased in circMagi1^−/−^ mice compared to wild-type (WT) mice (Fig. S3). *P. aeruginosa* was used to establish a lung infection model in wild-type (WT) and circMagi1^−/−^ mice. Twenty-four hours post-infection, whole-lung tissues were collected for cytometry by time-of-flight (CyTOF) analysis (Fig. 3A). In the lung tissue, 18 immune cell subsets were identified, which were categorized into five main types: T cells, B cells, NK cells, macrophages, and neutrophils (Fig. 3B). Neutrophils comprised the large proportion of these immune cells, followed by macrophages and T cells. Further statistical analysis revealed that in circMagi1^−/^− mice, the number of T cells, B cells, NK cells, and macrophages was significantly increased, while the proportion of neutrophils decreased (Fig. 3C; Fig. S4A). This change reflects a remodeling of immune cell composition and is consistent with the elevated baseline inflammation (Fig. S3) and impaired bacterial clearance observed in circMagi1^−/−^ mice (Fig. 3E), suggesting that circMagi1 deficiency may lead to a dysregulated antibacterial immune response, in which neutrophils are insufficiently recruited or maintained despite heightened inflammation, thereby compromising host antibacterial defense. To validate the CyTOF findings, we conducted additional *in vivo* experiments. Following lung infection, single-cell suspensions were prepared from mouse lung tissues for cytometry analysis. Specific markers were used to identify immune cell types: CD3 for T cells, CD19 for B cells, NK1.1 for NK cells, CD11b and F4/80 for macrophages, and CD11b and Ly6G for neutrophils (Fig. S4B). Consistent with the CyTOF results, the proportions of T cells, B cells, and macrophages were significantly higher in circMagi1^−/−^ mice than in WT mice, while the proportion of neutrophils was lower (Fig. 3D; Fig. S4C). Taken together, circMagi1 deletion led to marked alterations in lung immune cell composition, characterized by increased proportions of T cells, B cells, NK cells, and macrophages among CD45^+^ cells, alongside a decreased neutrophil proportion (Fig. 3C and D; Fig. S4). This immune remodeling correlates with impaired bacterial clearance (Fig. 3E), further highlighting the critical role of circMagi1 in maintaining pulmonary immune homeostasis and an effective antibacterial response.

### circMagi1 binds to the G3BP2 protein and enhances its stability

**Fig 4:**
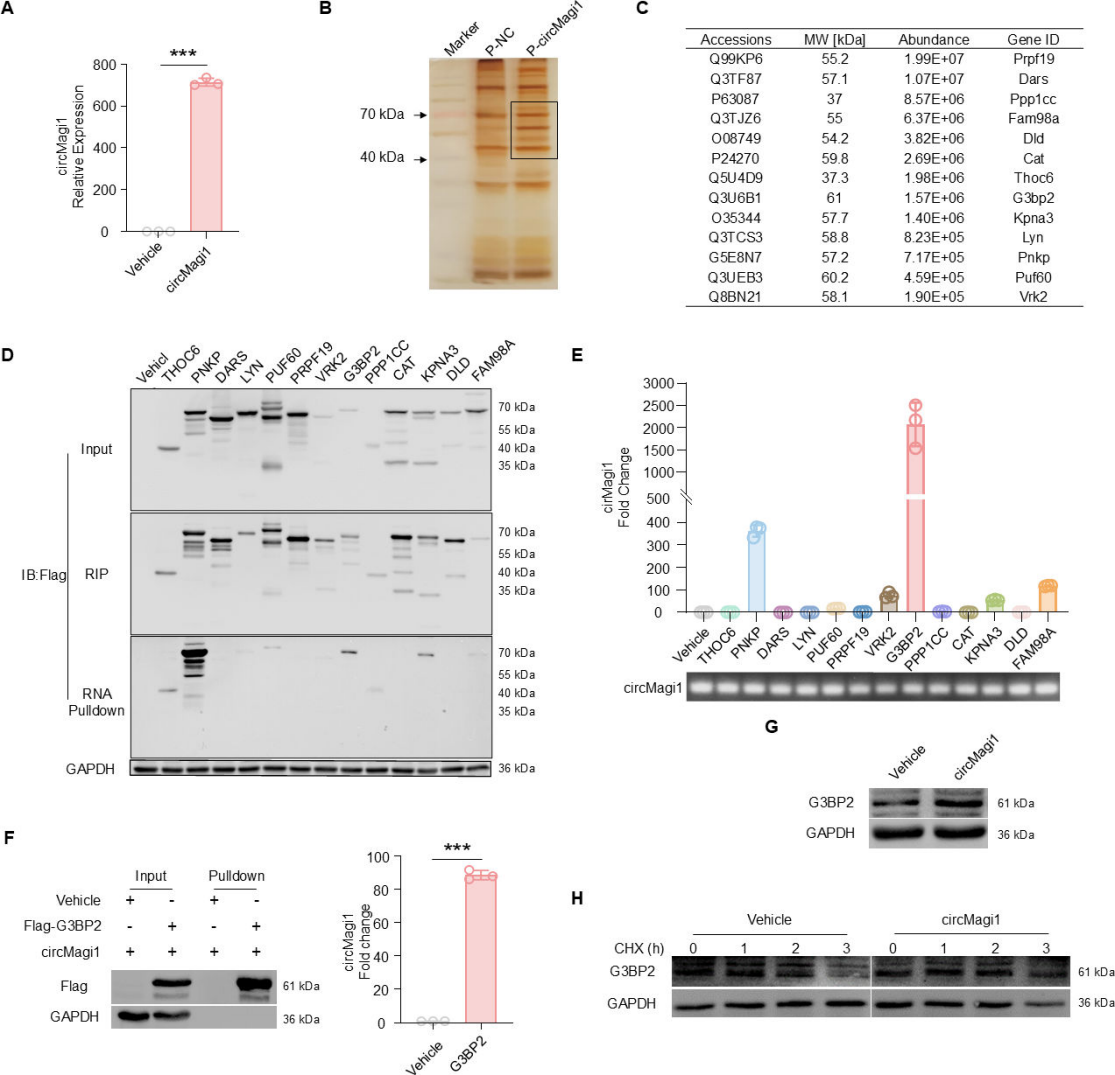
CircMagi1 binds to the G3BP2 protein and enhances its protein stability. (**A**) qPCR detection of circMagi1 overexpression in iBMDMs. (**B**) Silver staining showing proteins bound to circMagi1. The circMagi1 experiment revealed obvious specific bands at 40–70 kDa. iBMDMs overexpressing circMagi1 were lysed, and circMagi1 and its interacting proteins were enriched via streptavidin magnetic beads conjugated with biotinylated NC (negative control) or circMagi1 probes. (**C**) Preselected target proteins. (**D, E**) Validation of circMagi1-protein interactions by RNA pulldown and RIP assays. circMagi1 and Flag-tagged target proteins were co-expressed in 293T cells for 48 h. Western blot was performed on 90% of the pulldown lysates and 10% of the RIP lysates to detect associated proteins. qPCR was performed on 10% of the pulldown and 90% of the RIP lysates to detect circMagi1. The bottom panel in panel **E** shows qPCR products for circMagi1 from input samples. (**F**) RNA pulldown and RIP verified the interaction of circMagi1 with G3BP2. G3BP2 and circMagi1 were overexpressed in 293T cells. After 48 h, their interaction was detected as detailed in panels **D and E**. (**G**) Western blot analysis of the effect of circMagi1 on G3BP2 protein expression. iBMDMs with or without circMagi1 overexpression were lysed, after which G3BP2 protein levels were detected. (**H**) Western blot analysis of the effect of circMagi1 on G3BP2 protein expression after CHX treatment at different infection time points. iBMDMs were treated with 10 μg/mL CHX. All data are presented as the mean ± SD. Statistical significance was assessed using unpaired *t* tests with Welch’s correction if necessary (**A, F**). ***P < 0.001. CHX, cycloheximide; iBMDM, immortalized bone marrow-derived macrophage; RIP, RNA immunoprecipitation.

circRNAs usually function as miRNA sponges, bind to RNA-binding proteins (RBPs), or serve as templates for translation (30). Our experiments showed that circMagi1 can bind to AGO2 (Fig. S5A), a core component of the RNA-induced silencing complex (RISC) that mediates miRNA-guided gene silencing, suggesting a potential role as a miRNA sponge (12). However, given the relatively short sequence of circMagi1 and the limited number of predicted miRNA binding sites, we focused on exploring other possible functions. To evaluate its translational potential, we examined the association between circMagi1 and the ribosomal protein S6 (RPS6). RNA-binding protein immunoprecipitation (RIP) experiments revealed no detectable interaction (Fig. S5B), suggesting that circMagi1 is unlikely to be translated. These findings led us to investigate whether circMagi1 primarily exerts its function through protein interactions. We thus turned our attention to identifying specific circMagi1-binding proteins using RNA pulldown and RIP approaches. Lysates from iBMDMs overexpressing circMagi1 (Fig. 4A) were incubated with control or circMagi1 probes (Fig. S5C). The proteins pulled down by these probes were separated via 10% SDS-PAGE and visualized using silver staining (Fig. S5D). Notably, the circMagi1 probe specifically enriched protein bands in the 40–70 kDa range (Fig. 4B). Differentially expressed protein bands were subjected to mass spectrometry analysis, and candidate targets were selected for validation based on their abundance, molecular size, and functional relevance (Fig. 4C). Among these candidates, G3BP2, a member of the Ras-GTPase-activating protein SH3-domain-binding protein family, is an RNA-binding protein that plays a crucial role in stress granule assembly and the regulation of mRNA stability (31). Moreover, G3BP2 has been implicated in inflammatory signaling and host defense responses (31). Pulldown and RIP assays confirmed that circMagi1 binds to multiple proteins to varying extents, with G3BP2 showing the strongest binding affinity (Fig. 4D through F). To determine the specific domain of G3BP2 required for binding to circMagi1, truncated G3BP2 mutants were constructed. The results demonstrated that circMagi1 binding requires the full-length G3BP2 protein (Fig. S5E). Since circRNAs typically influence transcriptional or posttranscriptional regulation or modulate protein activity after acting as protein sponges, we assessed whether circMagi1 affects G3BP2 protein expression. The findings revealed that circMagi1 increases G3BP2 protein levels (Fig. 4G) without significantly altering its RNA levels (Fig. S5F). Consistent with this, G3BP2 protein levels showed a decreasing trend in lung tissues of uninfected circMagi1^−/−^ mice (Fig. S5G), providing *in vivo* evidence that circMagi1 promotes G3BP2 protein expression. Furthermore, treatment with cycloheximide (CHX) showed that circMagi1 extends the half-life of G3BP2, thereby enhancing its stability (Fig. 4H).

### circMagi1 increases G3BP2 protein stability by suppressing K48 ubiquitination

**Fig 5:**
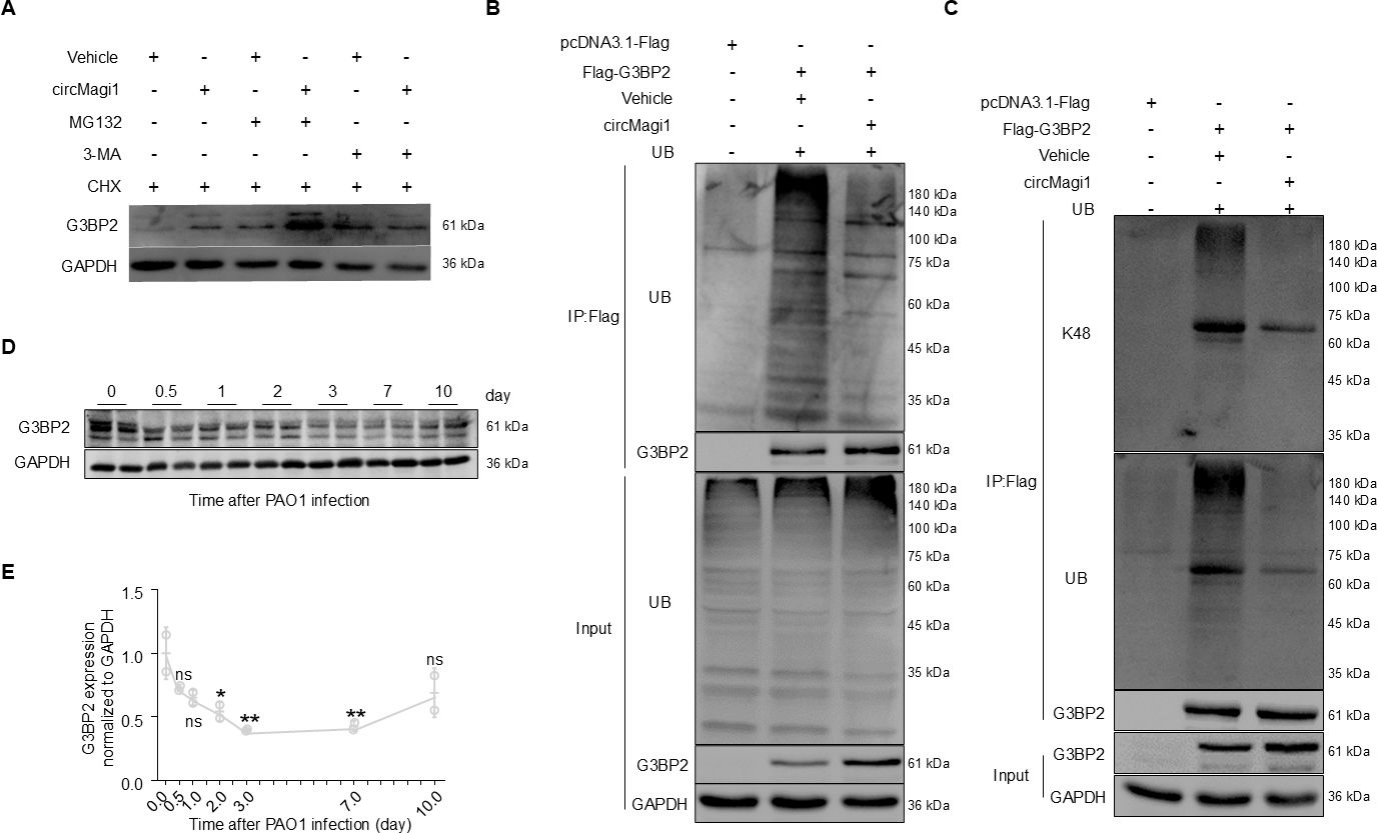
CircMagi1 affects G3BP2 protein stability by inhibiting K48 ubiquitination. (**A**) Western blot analysis of the effect of circMagi1 on G3BP2 protein expression after treatment. iBMDMs were treated with 10 μg/mL CHX, 10 μM MG132, or 10 μM 3-MA. (**B**) Western blot analysis of the effect of circMagi1 on G3BP2 protein ubiquitination. The target proteins and circMagi1 were overexpressed in 293T cells, and interactions were detected after 48 h. (**C**) Western blot analysis of the effect of circMagi1 on K48-linked ubiquitination of the G3BP2 protein. The target proteins and circMagi1 were overexpressed in 293T cells, and interactions were detected after 48 h. (**D**) Western blot analysis of G3BP2 in mouse lungs at different infection time points. There were four to five mice in each group (two mice were randomly selected), and each mouse was infected with 3 × 10^6^ CFU of PAO1. (**E**) Quantitative statistics for the results shown in panel **D**. All data are presented as the mean ± SD. Statistical significance was assessed using one-way ANOVA (**E**) with comparison to the 0 h control. ns, not significant; **P < 0.01 and *P < 0.05. 3-MA, 3-methyladenine; CHX, cycloheximide; iBMDM, immortalized bone marrow-derived macrophage.

To further explore how circMagi1 enhances protein stability, we treated cells with MG132, a proteasome inhibitor, and 3-methyladenine (3-MA), an autophagy inhibitor. The results showed that circMagi1 increases G3BP2 protein stability via the proteasome system (Fig. 5A). The ubiquitin-proteasome system is the primary pathway for protein degradation. To assess this, we examined the ubiquitination of G3BP2 and found that circMagi1 significantly reduces its overall ubiquitination level (Fig. 5B). Since K48-linked ubiquitination is related to proteasome degradation, we specifically measured K48 ubiquitination of G3BP2. The results showed that circMagi1 markedly decreases the K48 ubiquitination level of G3BP2 (Fig. 5C). Furthermore, we analyzed G3BP2 protein expression in mouse lung tissue following *P. aeruginosa* infection. The expression of G3BP2 decreased initially and then recovered during the later stages of infection (Fig. 5D and E). Notably, circMagi1 expression was significantly downregulated at 24 h post-infection (Fig. 1B), while G3BP2 protein expression significantly decreased at 48 h. This temporal correlation further supports the role of circMagi1 in regulating G3BP2 protein stability. In conclusion, our findings suggest that circMagi1 enhances G3BP2 protein stability by suppressing K48-linked ubiquitination.

### circMagi1 regulates the macrophage inflammatory response through G3BP2

**Fig 6:**
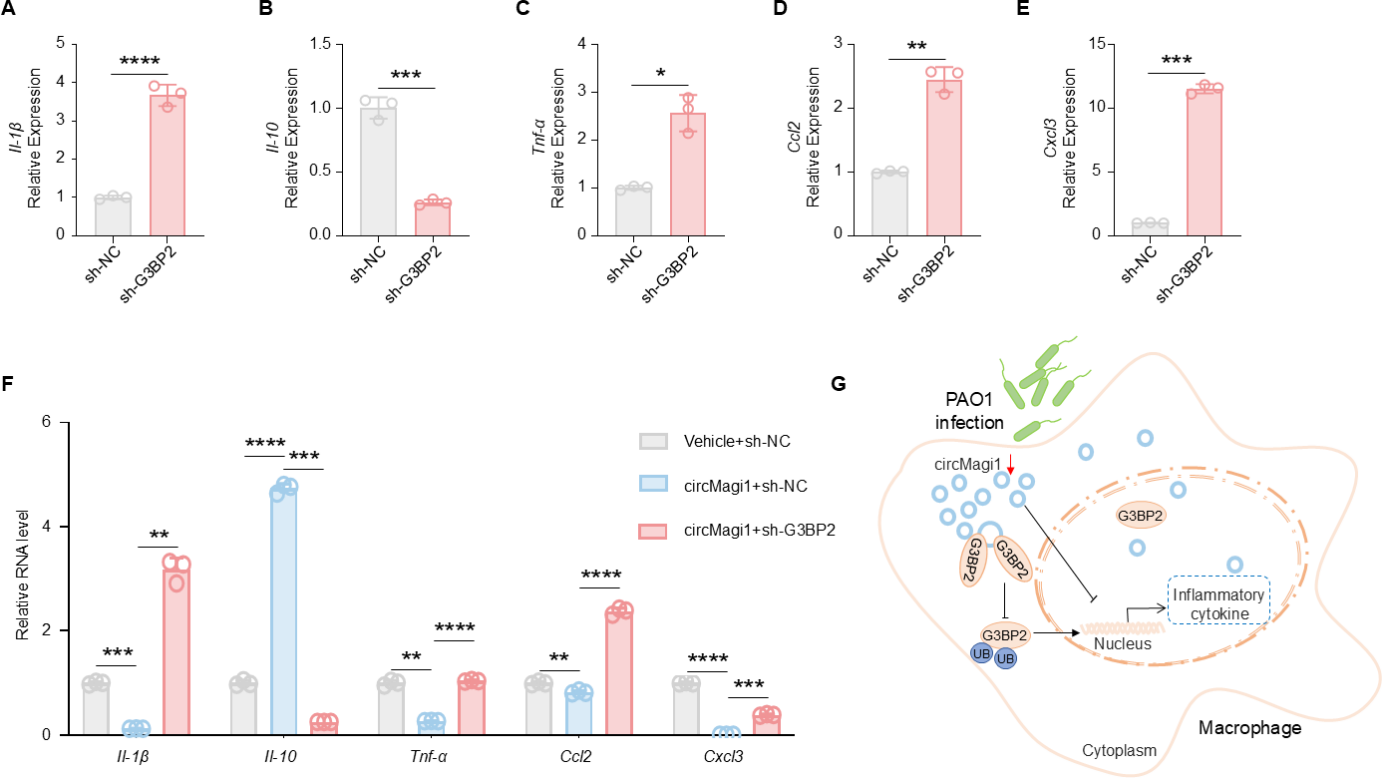
CircMagi1 regulates the expression of macrophage inflammatory factors through G3BP2. (**A‒E**) qPCR detection of *Il-1*β, *Il-10*, *Tnf-*α, *Ccl2*, and *Cxcl3* in sh-NC and sh-G3BP2 treated iBMDMs. The cells were treated with PAO1 for 6 h before analysis. (**F**) qPCR detection of *Il-1*β, *Il-10*, *Tnf-*α, *Ccl2*, and *Cxcl3* expression after overexpressing circMagi1 and RNA interference-based disruption of G3BP2 or a control in iBMDMs. All the experiments were performed after 6 h of PAO1 treatment (MOI = 20). (**G**) A proposed model depicting how the circMagi1-G3BP2 axis may contribute to the regulation of macrophage inflammation. All data are presented as the mean ± SD. Statistical significance was assessed using unpaired *t* tests with Welch’s correction if necessary (**A–F**). ns, not significant; ****P < 0.0001, ***P < 0.001, **P < 0.01, and *P < 0.05. iBMDM, immortalized bone marrow-derived macrophage.

To further explore the function of the G3BP2 protein, we constructed a G3BP2-specific RNA interference cell line via lentivirus infection and verified the interference efficiency via qPCR, western blot, and immunofluorescence (IF) (Fig. S6). Following this, the cells were treated with PAO1, and the levels of inflammatory factors and chemokines were measured. RNA interference-mediated reduction of G3BP2 led to varying degrees of upregulation of the proinflammatory factors, including Il-1β, Tnf-α, Ccl2, and Cxcl3. In particular, the expression of the chemokine Cxcl3 increased by over 10-fold, while the expression of the anti-inflammatory cytokine Il-10 was significantly reduced (Fig. 6A through E). These results suggest that G3BP2 plays a role in suppressing inflammatory responses during PAO1 stimulation, a function resembling that of circMagi1. Notably, G3BP2 knockdown did not affect circMagi1 expression (Fig. S5H), suggesting that G3BP2 is unlikely to regulate circMagi1 expression in a feedback manner. Given that circMagi1 binds to G3BP2 and enhances its stability, we explored whether the anti-inflammatory function of circMagi1 depends on G3BP2.

We overexpressed circMagi1 while reducing G3BP2 expression in iBMDMs. Following PAO1 treatment, qPCR was used to assess the levels of inflammatory factors. Overexpression of circMagi1 significantly reduced the levels of proinflammatory cytokines, such as Il-1β, Tnf-α, Ccl2, and Cxcl3, while significantly increasing the expression of the anti-inflammatory cytokine Il-10. However, when G3BP2 expression was reduced via RNA interference, the cytokine changes induced by circMagi1 overexpression were reversed. Proinflammatory cytokine levels increased, and Il-10 expression decreased (Fig. 6F). These findings suggest that circMagi1 regulates the expression of inflammatory factors through the G3BP2 protein.

## DISCUSSION

Studies have shown that circRNAs are involved in the development of various health issues, including tumors, autoimmune diseases, cardiovascular diseases, and neurological diseases (14, 32). Many circRNAs have also been identified as potential disease biomarkers (33). However, the role of circRNAs in the host immune response to bacterial infection remains largely unexplored. Identifying novel immune regulatory molecules could facilitate the discovery of new diagnostic and therapeutic biomarkers.

Bacterial infections trigger host immune activation as a defense mechanism, but excessive immune activation can lead to severe tissue damage (34). Thus, negative regulatory factors are essential for maintaining immune response balance. However, the identification of negative regulatory factors during bacterial infections remains incomplete. In this study, we observed a significant decrease in circMagi1 expression in a mouse model of *P. aeruginosa* lung infection. The expression trend of circMagi1 exhibited a potential correlation to a certain extent with the immune activation during bacterial infection (25). Similarly, circMagi1 was significantly downregulated in the BALF of patients following bacterial infection. Based on these findings, we hypothesized that circMagi1 is involved in the host immune response process. Typically, the host initiates an immune response by producing inflammatory cytokines and modulating the activation state of immune cells (27). To investigate this process, we constructed a macrophage model overexpressing circMagi1 and a circMagi1 knockout mouse model. Transcriptome sequencing and CyTOF analysis of lung tissues revealed that circMagi1 inhibits host immune function. Given that circRNAs typically function by binding to proteins (30), we employed a circMagi1 probe to pull down its protein binding partners for mass spectrometry identification. Among the candidate targets, G3BP2 was selected for further investigation. Previous studies have shown that G3BP2 regulates inflammatory cytokine expression through NF-κB signaling (35) and modulates the transcription of antiviral and proinflammatory factors in innate immunity (36, 37). In addition, knockdown of G3BP2 has been reported to suppress cell proliferation while enhancing inflammatory gene expression (38). In our study, circMagi1 was found to bind G3BP2 and increase its stability by inhibiting ubiquitination, thereby modulating cytokine expression in macrophages (Fig. 6G). These findings provide new insight into the context-dependent role of G3BP2 in innate immunity. Under conditions of PAO1 infection in macrophages, G3BP2 is more likely to function as a negative regulator of inflammatory cytokine expression, as circMagi1-mediated stabilization of G3BP2 was associated with reduced inflammatory cytokine levels. Mechanistically, given that G3BP2 has been reported to modulate NF-κB-related signaling pathways and transcriptional programs (35–37), increased G3BP2 stability may limit excessive inflammatory signaling. Although the precise molecular mechanisms remain to be fully elucidated, these observations support the involvement of the circMagi1-G3BP2 regulatory relationship in macrophage inflammatory responses.

Notably, even in the presence of MG132, circMagi1 significantly increased G3BP2 protein levels, suggesting that its regulatory effect is not solely dependent on the inhibition of proteasomal degradation. It is possible that MG132 does not completely inhibit proteasome activity under the experimental conditions (39), and circMagi1 may further attenuate the residual proteasomal activity. Additionally, although the addition of 3-MA did affect G3BP2 levels, its impact was less pronounced compared to the changes observed under vehicle or circMagi1 overexpression conditions. This suggests that autophagy might contribute to G3BP2 stability to some extent but is unlikely to be the dominant pathway. Instead, circMagi1 may stabilize G3BP2 through enhanced translation or modulation of alternative protein degradation pathways. Although the exact mechanisms remain to be fully elucidated, our findings underscore the complex role of circMagi1 in regulating G3BP2 stability and provide a foundation for further investigations. This study highlights circMagi1 as a negative regulatory factor that participates in the host immune response to bacterial infection by modulating macrophage inflammatory cytokine expression through the G3BP2 protein.

At the same time, we note several limitations in this work. circRNAs usually function as protein and miRNA sponges (40–42). In this study, because the sequence of circMagi1 is short, we did not investigate its function as a miRNA sponge. However, verification of the interaction between the circMagi1 and AGO2 proteins (Fig. S5A) revealed that circMagi1 functions as a miRNA sponge (12). Therefore, we focused on the protein sponge function of circMagi1 and did not investigate its miRNA sponge function in detail. However, miRNAs such as miR-301b and miR-302b play critical roles during *P. aeruginosa* infection by regulating inflammatory cytokine expression, macrophage phagocytosis, and neutrophil recruitment (43, 44). CircMagi1 may act as a miRNA sponge, sequestering these functional miRNAs, modulating their target genes, and indirectly affecting inflammation and immune cell function. Future studies are required to explore this potential mechanism. Moreover, due to the fact that this study only performed gel excision and mass spectrometry analysis on the differentially expressed bands within the 40–70 kDa region that exhibited clear and stable differences across repeated experiments, other potentially differentially expressed bands on the gel were not covered, which may have overlooked some relevant proteins. Additionally, mass spectrometry revealed other potential circMagi1-binding targets, which also warrant further investigation. Additionally, while macrophages were chosen for this study, the role of circMagi1 in other lung cell types (7), which collectively contribute to the host immune defense during bacterial infections, remains unexamined.

In summary, this study identifies circMagi1 as a circRNA significantly downregulated during *P. aeruginosa* lung infection. Acting primarily as a negative regulator of immune activation, circMagi1 maintains immune balance by modulating the macrophage cytokine signaling axis through its regulation of G3BP2. These findings provide new insights into the role of circRNAs in bacterial infections.

## MATERIALS AND METHODS

### Bacterial strains and cell lines

Wild-type *P. aeruginosa* PAO1 (WT PAO1) was donated by the S. Lory Laboratory, Harvard Medical School, and was routinely cultured in Luria-Bertani broth with shaking at 37°C (220 rpm).

293T cells (NCACC, Cat# SCSP-502) were obtained from the Cell Bank of the Shanghai Institutes for Biological Sciences (Shanghai, China), and the iBMDM cell line was donated by the laboratory of Dr. Shao Feng at the National Institute for Biological Sciences (Beijing, China). 293T cells and iBMDM cells were cultured in DMEM (Cytiva, USA). The cell culture medium was supplemented with 50 U/mL penicillin and streptomycin (Gibco, USA) and 10% fetal bovine serum (PAN, Germany). All the cells were cultured in a humidified incubator at 37°C with 5% CO_2_.

### Mice

Eight-week-old female C57BL/6 mice were purchased from Beijing Huafukang Bioscience Co., Ltd. (Beijing, China). circMagi1 knockout mice (circMagi1^−/−^) were purchased from Sayer Biotechnology Co., Ltd. (Shenzhen, China). To construct the circMagi1^−/−^ model, two guide RNAs (gRNAs) targeting the intronic Alu repeat sequence between exons 4 and 5 of the Magi1 gene were designed: gRNA1 (matching reverse strand of gene), GAAGAATAGTTGGTTGTCGGTGG; gRNA2 (matching reverse strand of gene): AGCCCTTATTATTTTGCAAGAGG.

These gRNAs and Cas9 mRNA were co-injected into fertilized mouse embryos to generate targeted knockout offspring through a specific deletion of the intronic sequence. The deletion disrupted the production of circMagi1 by removing the intronic sequence necessary for its circularization, while preserving exon sequences required for normal splicing of the linear Magi1 transcript, ensuring its expression remained unaffected. All the mice were housed in a specific pathogen-free facility at the State Key Laboratory of Biotherapy, Sichuan University.

### Patient alveolar lavage fluid

Patient alveolar lavage fluid was collected by the Department of Respiratory and Critical Care Medicine, West China Hospital, Sichuan University.

### Infection models

The PAO1 strain was routinely cultured in Luria-Bertani broth with shaking at 37°C (220 rpm), and the bacterial mixture was resuspended and diluted in sterile saline. The optical density (OD) of the bacterial mixture was subsequently measured via a UV spectrophotometer, and the bacterial concentration was calculated as 1 OD = 8.8 × 10^8^ CFU/mL.

For the cellular infection model, the required bacterial amount was calculated and added to DMEM (without serum or antibiotics). The bacterial mixture was then used to replace the original medium for cell infection. After 1 h of infection, the bacterial mixture was discarded and replaced with DMEM (with serum, antibiotics, and 100 μg/mL gentamicin) for continued incubation. The cells were collected at specified time points based on the experimental requirements. The MOI was 20 for all experiments unless otherwise stated.

For the mouse lung infection model, the required bacterial volume was calculated to be 50 µL per mouse; 50 µL of PAO1 was introduced into the oropharyngeal cavity of each mouse via a tongue-root drop until the solution was completely aspirated into the lungs. The mice were sacrificed at the desired time point after infection, and lung tissue was collected for subsequent experiments.

### Plasmids and primers

The gene overexpression vectors used were pMax cloning, pLVX, and Flag-tagged pcDNA3.1. The RNA interference vector used was pLKO.1. psPAX2 and pMD2G were used as helper plasmids for viral packaging. Gene sequences were obtained from the NCBI and circBase databases. The templates used for gene amplification were all obtained from wild-type mouse lung tissues. For the overexpression plasmids, homologous recombination primers were designed according to the vector cleavage sites and CDSs of the amplified genes, which were synthesized by Tsingke Biotech, and the overexpression vectors were then constructed via the Trelief SoSoo Cloning Kit Ver.2 (Tsingke, China). For the overexpression of circMagi1, we inserted exon 3 into exon 4 of Magi1 between the upstream 1,000-bp genomic sequence and the downstream 200-bp genomic sequence, fused it with the reverse repeat sequence of the upstream 800-bp genomic sequence to obtain the circMagi1 sequence, and then cloned the sequence into the pMAX and pLVX vectors via homologous recombination. For gene disruption, we ligated shRNAs targeting G3BP2 (sh1: 5′-TTCGAGGAGAAGTACGTTTAA-3′; sh2: 5′-CTCTGACAACCGGAGAATAAT-3′) into the pLKO.1 vector. The sequences of the qPCR primers used are listed in Table S1.

### Cytometry by time-of-flight (CyTOF)

A mouse lung infection model was established, and the mice were sacrificed 24 h post-infection to collect whole lung tissues. Lung tissues from two to three mice were pooled into one sample, minced into small pieces, and digested with 4.5 mL Lung Dissociation Kit (Miltenyi, Germany) at 37°C for 1 h with gentle shaking. The resulting cell suspensions were filtered through a 70 μm strainer, followed by red blood cell lysis and washing with FACS buffer (1× PBS with 0.5% BSA). The cells were collected by centrifugation and incubated with 100 μL of 250 nM cisplatin (Fluidigm, USA) to exclude dead cells. After blocking Fc receptors, the cells were stained with a cocktail of 42 surface and intracellular antibodies (Table S4) and labeled with unique Palladium-based mass tag barcodes. The barcoded samples were washed with deionized water, resuspended in 20% EQ beads (Fluidigm, USA), and analyzed on a Helios Mass cytometer (Fluidigm, USA). The generated data files were debarcoded using a doublet-filtering scheme, normalized through bead-based calibration, and manually gated to retain live, single immune cells. Cell clustering and phenotype annotation were performed using the Phenograph algorithm, while t-SNE dimensionality reduction was applied to visualize high-dimensional data. Statistical analysis of immune cell populations was conducted using FlowSOM to assess the frequencies of distinct cell subsets.

### Lung tissue dissociation and flow cytometry

Mouse lung tissues were collected and placed in a MACS C-tube (Miltenyi, Germany), 4 mL of DMEM containing 10% FBS and 0.5 mg/mL collagenase type I/IV was added, and the lung tissues were dissociated via the MACS Octo Dissociator with Heaters following the manufacturer’s protocol (Miltenyi, Germany) for lung tissue dissociation. After dissociation, the single-cell suspensions were passed through a 70 μm filter, and red blood cells were lysed with red blood cell lysis buffer (Solarbio, R1010). The resulting cells were resuspended and counted and then stained with the following fluorescence staining antibodies from BioLegend: CD45 (103105, 1:100), CD45 (339203, 1:100), CD11b (101228, 1:100), Ly-6G (127617, 1:100), F4/80 (123116, 1:100), CD3 (100312, 1:100), NK1.1 (108708, 1:100), and CD19 (152407, 1:100). Flow cytometry was performed on a FACS Caliber (BD Biosciences) or Novocyte (Agilent Biosciences).

### RNase R treatment

RNAs extracted from the lung tissues were split into two aliquots: one for RNase R digestion and the other for the control with digestion buffer only. For RNase R digestion, 2 μg of total RNA was incubated for 20 min at 37°C with 5 U/μg RNase R (Epicenter Technologies, USA); for the control, 2 μg of total RNA was incubated for 20 min at 37°C.

### Isolation of cytoplasmic and nuclear RNA

A total of 3 × 10^6^ cells were prepared and transferred to 1.5 mL centrifuge tubes. The cells were washed with PBS to remove residual culture medium and processed using the Cytoplasmic & Nuclear RNA Purification Kit (Norgen Biotek, Canada) to separate cytoplasmic and nuclear RNA. Purified RNA was reverse-transcribed directly and analyzed by qPCR.

### Inhibitor treatment

Cycloheximide (MCE, USA) was dissolved in DMSO to a liquid concentration of 50 mg/mL for later use. iBMDMs were exposed to DMEM containing 10 μg/mL cycloheximide for 1, 2, or 3 h. The cells were harvested at indicated times. MG132 (Selleck, USA) was dissolved in DMSO to a liquid concentration of 10 mM for later use. iBMDMs were exposed to DMEM containing 10 μM MG132 for 2 h. The cells were harvested at indicated times. 3-Methyladenine (Selleck, USA) was dissolved in DMSO to a liquid concentration of 10 mM for later use. iBMDMs were exposed to DMEM containing 10 μM 3-MA for 2 h. The cells were harvested at indicated times.

### RNA fluorescence *in situ* hybridization (FISH)

The cells were placed into a 24-well plate, and iBMDMs were then seeded for overnight incubation. The cells were analyzed via a FISH kit (RiboBio, China) in accordance with the manufacturer’s instructions. Cy3-labeled circMagi1 and 18S rRNA probes were synthesized by RiboBio (Guangzhou, China). All images were acquired with an A1 HD25 confocal fluorescence microscope (Nikon, Japan).

### Immunofluorescence (IF)

The iBMDMs were seeded in 24-well plates overnight. The cells were fixed with 4% paraformaldehyde (Biosharp, China) for 15 min and treated with a tissue permeabilizer (Beyotime, China) for 10 min. Then, the cells were blocked with goat serum (Zsbio, ZLI-9022) for 30 min at room temperature and incubated with a primary antibody overnight at 4°C. The cells were incubated with secondary antibody for 1 h at 37 °C in the dark, washed with PBST three times, and blocked with DAPI (Beyotime, China). All images were acquired with an A1 HD25 confocal fluorescence microscope (Nikon, Japan).

### RNA isolation and quantitative real-time PCR (qPCR) assays

Cytoplasmic and nuclear RNA were extracted via an RNA Purification Kit (Norgen Biotek, Canada). Total RNA was isolated from lung tissues or cells with TRIzol reagent (Invitrogen, USA). The RNA was reverse-transcribed into cDNA with the PrimeScript RT reagent Kit with gDNA Eraser (Takara, USA) and quantified with TB Green Premix Ex Taq II (Takara, USA) on a LightCycler 96 Real-time PCR System (Roche, Switzerland). The expression of GAPDH/β-actin was used as a control to calibrate the original mRNA concentrations in tissues and cells. Target gene expression was calculated via the 2^−ΔΔCT^ method.

### Western blot analysis

Proteins were extracted with RIPA lysis buffer, separated by 10% SDS-PAGE, transferred to PVDF membranes (Millipore, USA), and blocked with 5% skim milk powder at 37°C for 60 min. The PVDF membranes were incubated with primary antibodies at 4°C overnight and then incubated with appropriate HRP-labeled secondary antibodies. An enhanced chemiluminescence (ECL) system (Beyotime, China) was used to develop the film, and digitized images were captured via an iBright CL1500 (Invitrogen, USA). Primary antibodies against G3BP2 (Proteintech, USA) and GAPDH (Cell Signaling Technology, USA) were used according to the instructions.

### RNA pulldown assays

Biotinylated circMagi1 (bio: 5′-TTGTGCCTGTAAAGCTACTG-3′) and NC probes were synthesized by RiboBio (Guangzhou, China). The indicated cells were lysed with immunoprecipitation lysis buffer containing protease inhibitor (Thermo, USA) and RNase inhibitor (Beyotime, China), and the cell lysates were centrifuged to remove debris, and the supernatant was collected. The biotin-labeled probes were incubated with streptavidin-conjugated magnetic beads (NEB, USA) at 25°C for 30 min, and the beads were then incubated with the supernatant at 4°C for 4 h. Afterward, the beads were washed with IP buffer three times. Finally, 10% of the binding product was prepared for RNA extraction, and qPCR was used to detect the binding of the biotinylated circMagi1 probe to circMagi1, while the remaining 90% were identified via Western blotting or mass spectrometry.

### RNA immunoprecipitation (RIP) assays

The indicated cells were lysed with IP lysis buffer containing protease inhibitor and RNase inhibitor, the cell lysates were centrifuged to remove debris, and the supernatant was collected and incubated with anti-Flag antibody-conjugated beads at 4°C overnight. Then, the beads were washed with IP lysis buffer three times. Finally, 10% of the binding product was used for Western blot to verify the binding of the target protein to the antibody, while the remaining 90% were prepared as RNA samples for qPCR detection.

### Co-immunoprecipitation (co-IP)

The indicated cells were lysed with IP lysis buffer containing protease inhibitor, the cell lysates were centrifuged to remove debris, and the supernatant was collected. The supernatant was mixed with magnetic beads and incubated with antibodies at 4°C overnight. Then, the beads were washed with IP lysis buffer three times. Finally, the proteins were eluted from the magnetic beads for Western blot analysis.

### Statistical analysis

Statistical analyses were performed via GraphPad Prism 8.0 (GraphPad Software, USA). Student’s *t* test was used to assess the significance of differences. *P* < 0.05 was considered to indicate statistical significance (*P < 0.05, **P < 0.01, ***P < 0.001, and ****P < 0.0001). All values are expressed as the mean ± SD.

## Data Availability

The raw and processed RNA sequencing data sets have been uploaded to the NCBI SRA database (No. PRJNA1132811) and are publicly available.

## SUPPLEMENTAL MATERIAL

Fig. S1 mbio.03617-25-s0001.tif Identification and characterization of circMagi1.

Fig. S2 mbio.03617-25-s0002.tif Construction of circMagi1 knockout mice.

Fig. S3 mbio.03617-25-s0003.tif Cytokine expression in the lung tissues of circMagi1 knockout mice.

Fig. S4 mbio.03617-25-s0004.tif circMagi1 affects host immune function.

Fig. S5 mbio.03617-25-s0005.tif CircMagi1 binds to the G3BP2 protein and enhances its stability.

Fig. S6 mbio.03617-25-s0006.tif Determination of G3BP2 RNA interference efficiency.

Legends mbio.03617-25-s0007.docx Supplemental figure legends.

Supplemental Tables mbio.03617-25-s0008.docx Tables S1-S4.

ASM does not own the copyrights to Supplemental Material that may be linked to, or accessed through, an article. The authors have granted ASM a non-exclusive, world-wide license to publish the Supplemental Material files. Please contact the corresponding author directly for reuse.
