# Supplementary figures and images for: Circular RNA circMagi1 regulates the host immune response in respiratory Pseudomonas aeruginosa infection through G3BP2
Source: mBio. 2026 Mar 23;17(4):e03617-25. doi: 10.1128/mbio.03617-25 (PMC13059810; doi:10.1128/mbio.03617-25)

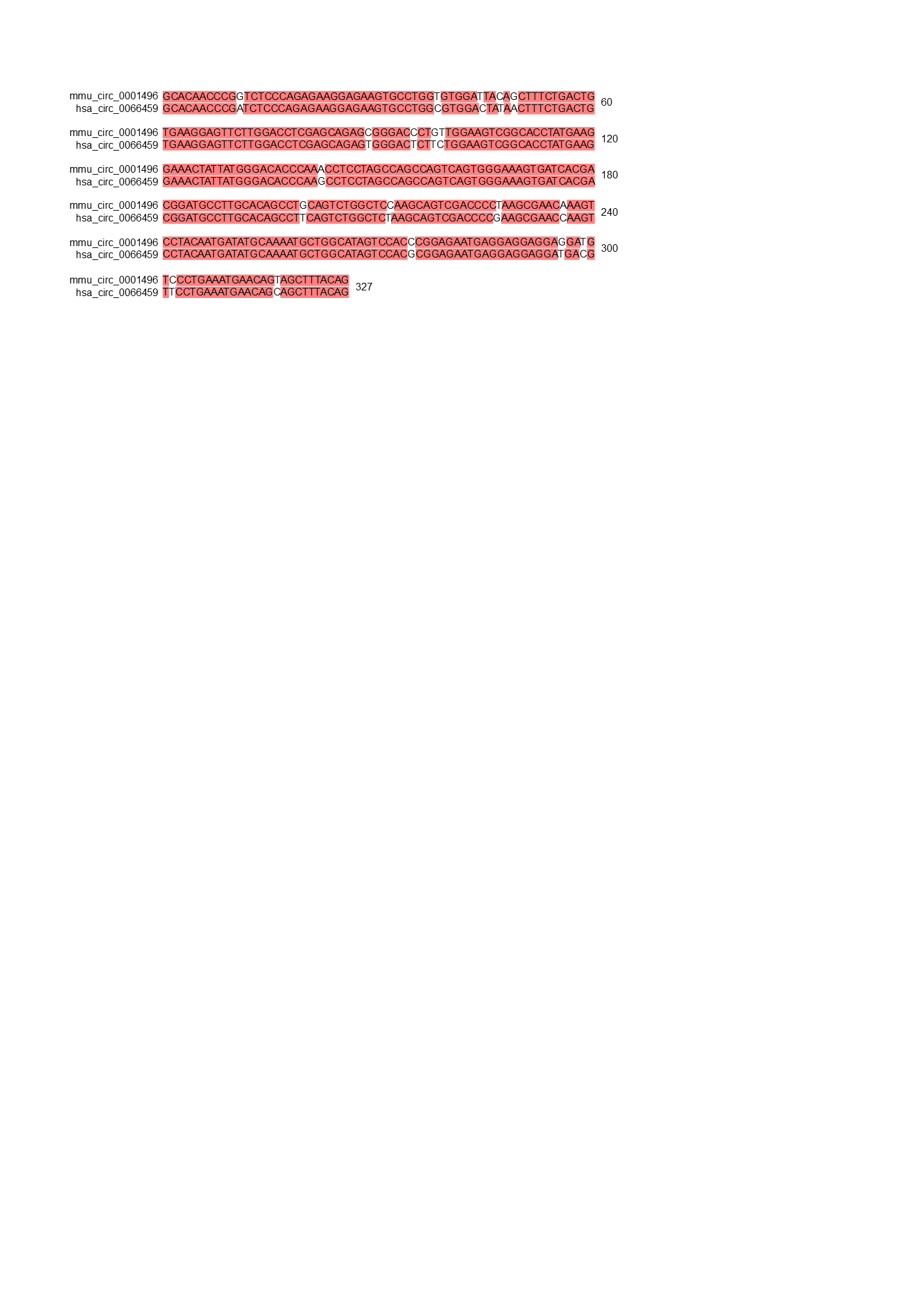

Supplement: Fig. S1 — Identification and characterization of circMagi1. [file mbio.03617-25-s0001.tif]

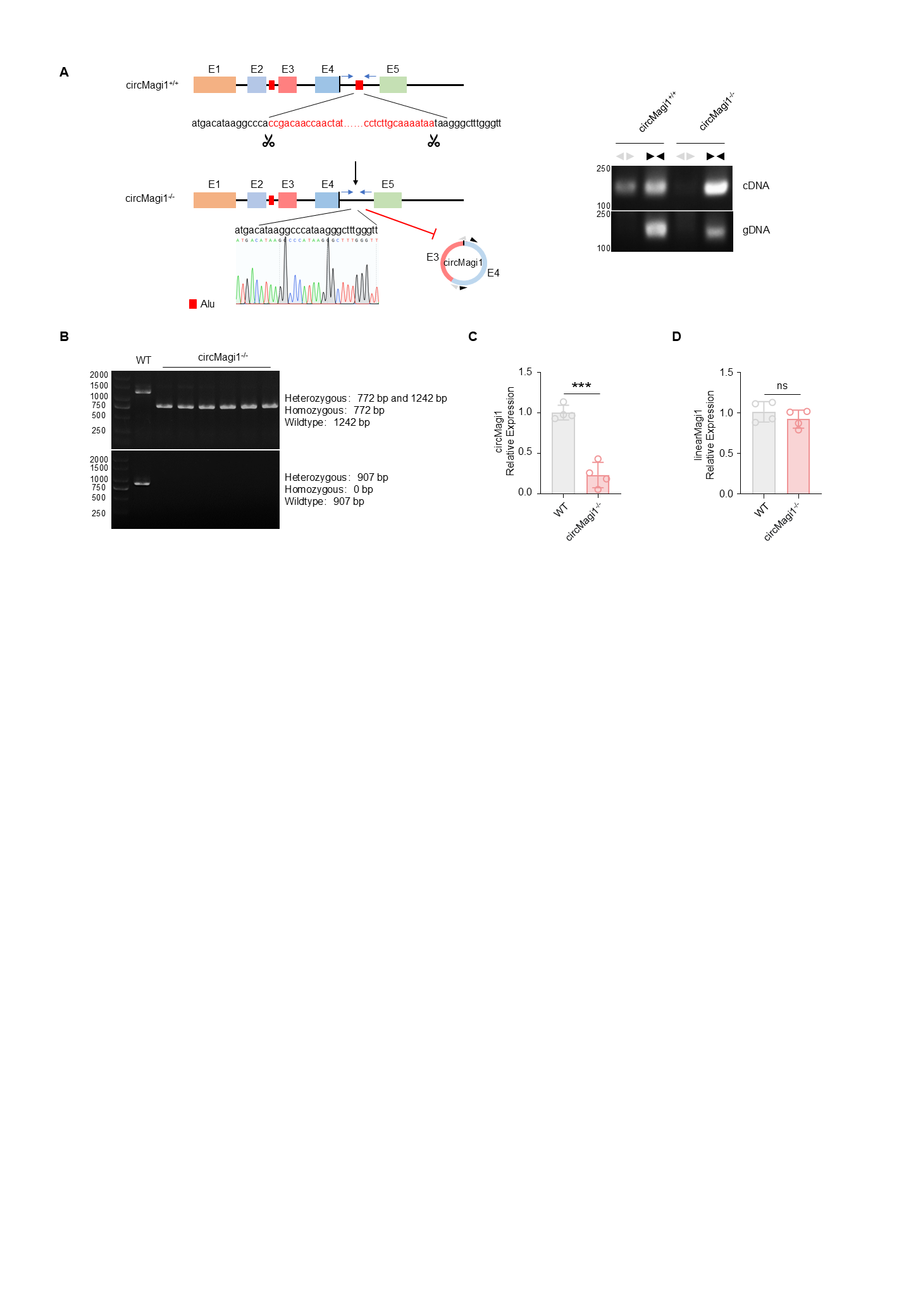

Supplement: Fig. S2 — Construction of circMagi1 knockout mice. [file mbio.03617-25-s0002.tif]

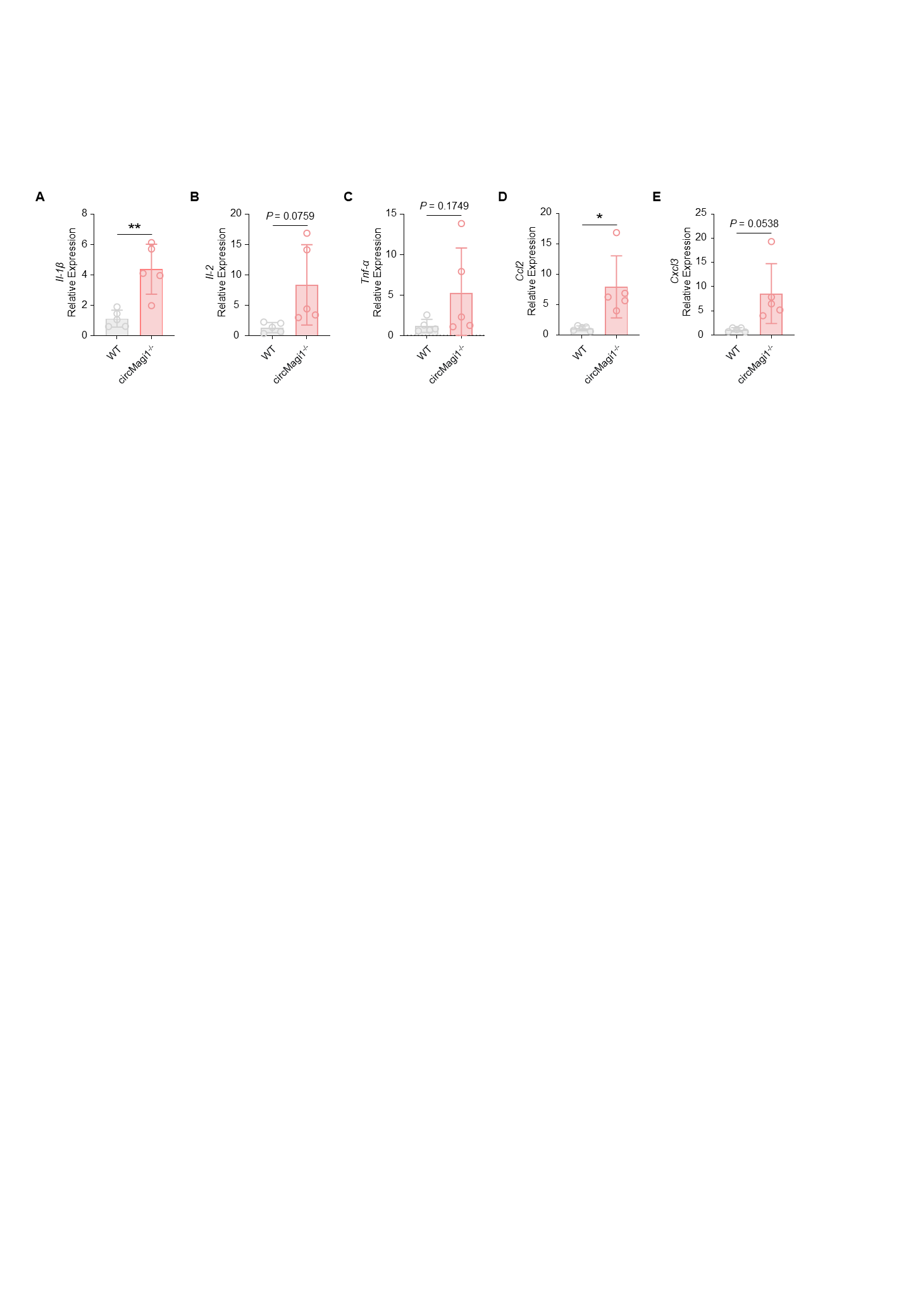

Supplement: Fig. S3 — Cytokine expression in the lung tissues of circMagi1 knockout mice. [file mbio.03617-25-s0003.tif]

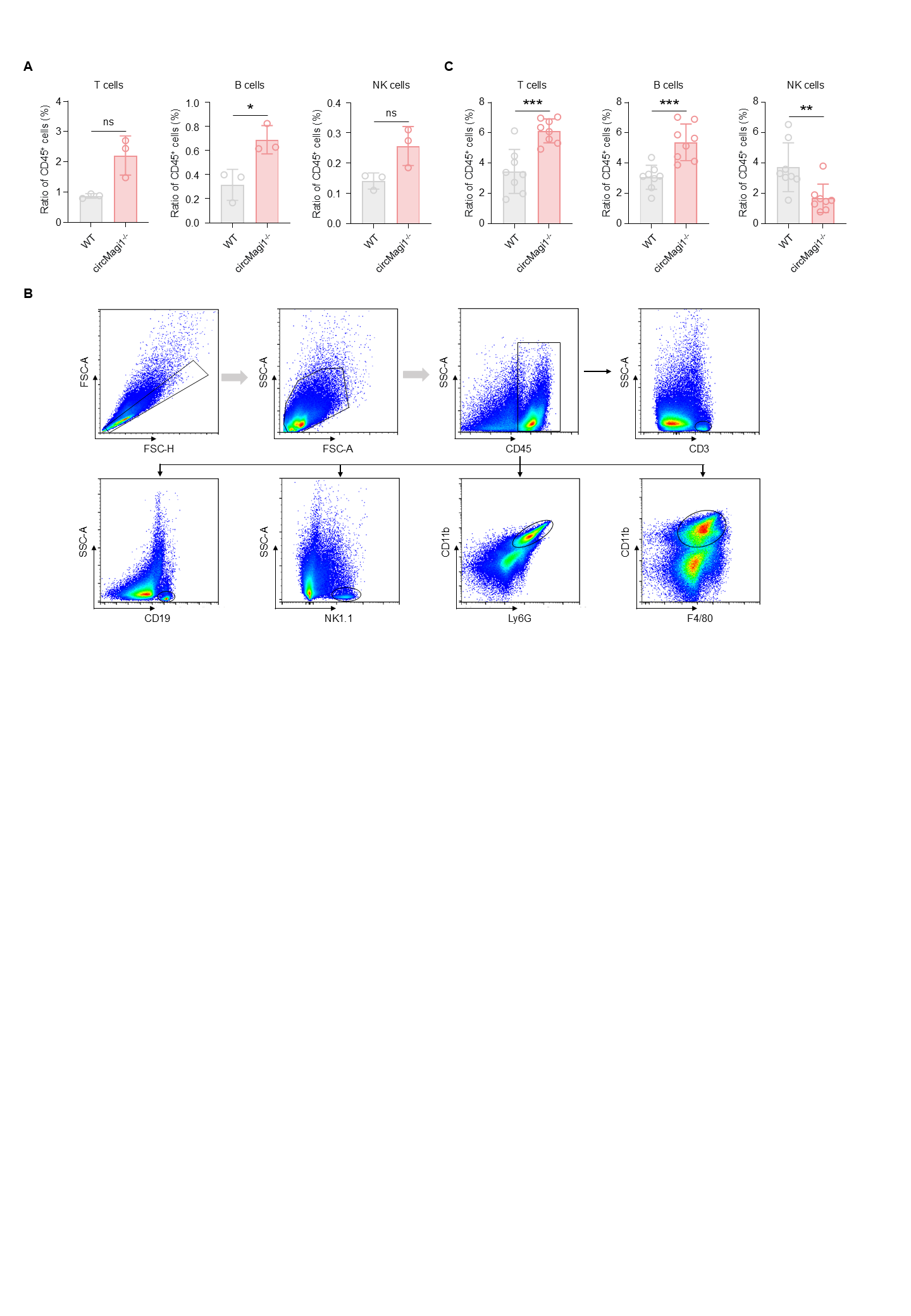

Supplement: Fig. S4 — circMagi1 affects host immune function. [file mbio.03617-25-s0004.tif]

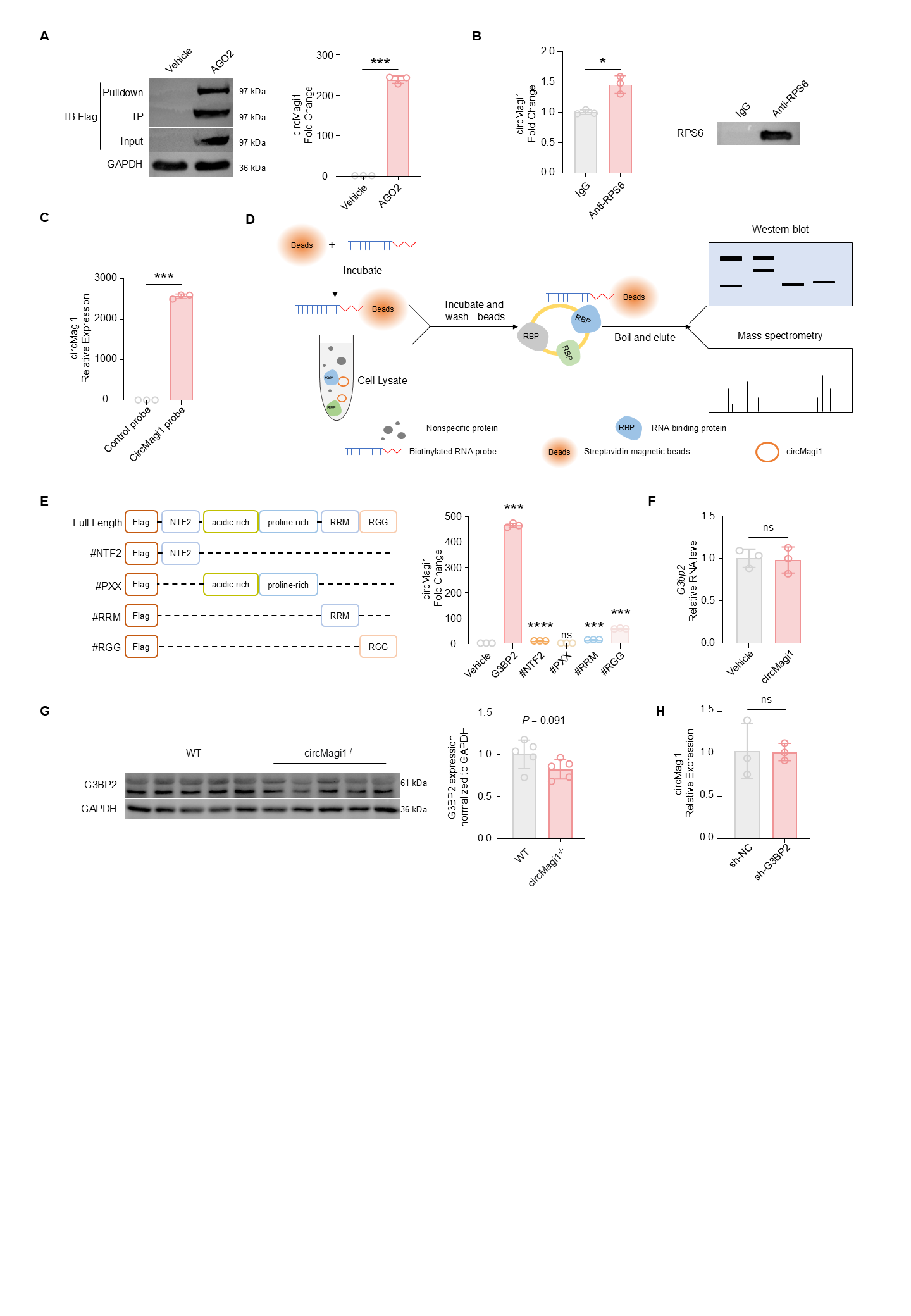

Supplement: Fig. S5 — CircMagi1 binds to the G3BP2 protein and enhances its stability. [file mbio.03617-25-s0005.tif]

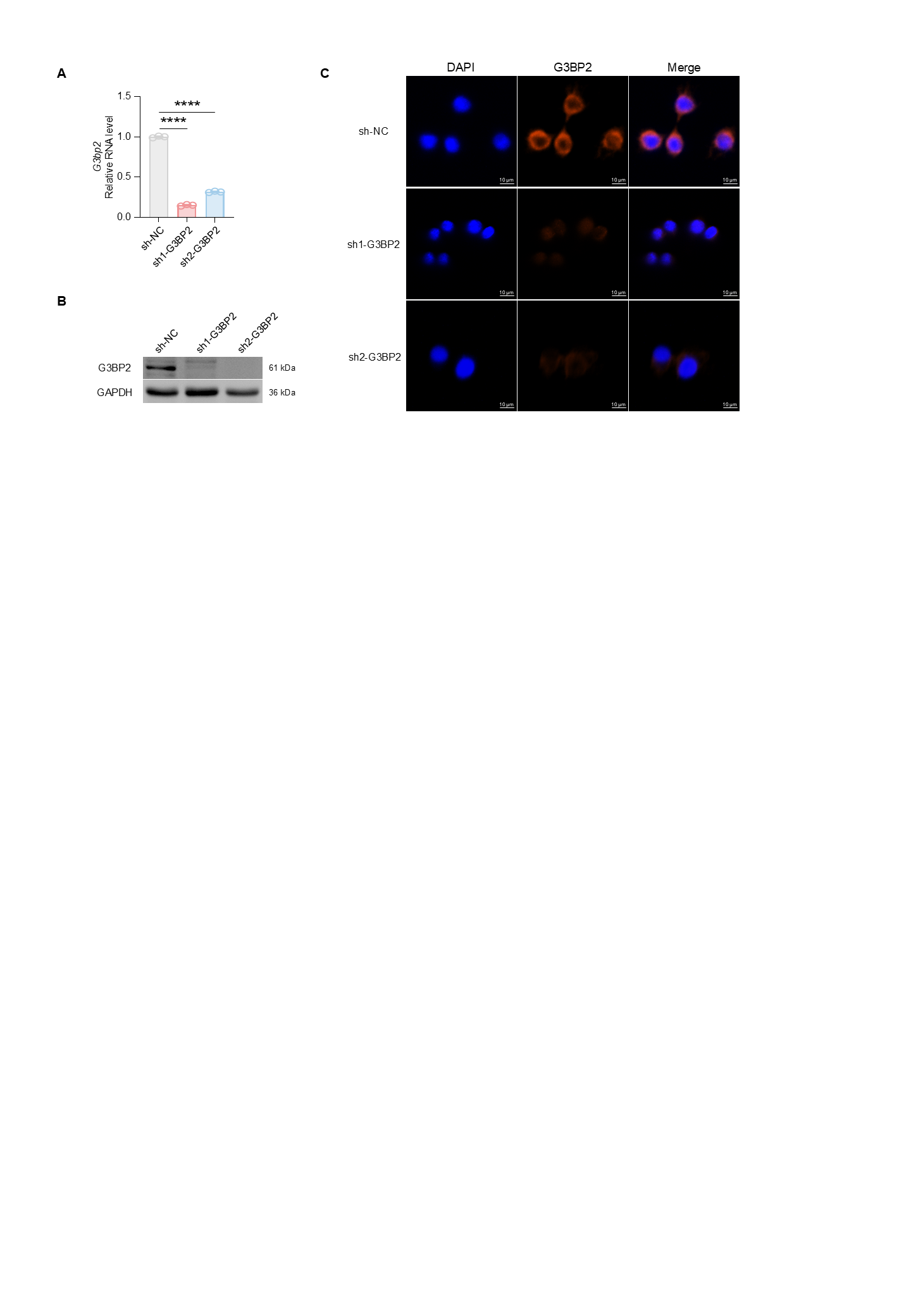

Supplement: Fig. S6 — Determination of G3BP2 RNA interference efficiency. [file mbio.03617-25-s0006.tif]
