# Supplementary material for: Circular RNA circMagi1 regulates the host immune response in respiratory Pseudomonas aeruginosa infection through G3BP2
Source: mBio. 2026 Mar 23;17(4):e03617-25. doi: 10.1128/mbio.03617-25 (PMC13059810; doi:10.1128/mbio.03617-25)
Supplement: Legends — Supplemental figure legends. [file mbio.03617-25-s0007.docx]

**Supplementary Figure Legends**

**Supplementary Fig. 1 | Identification and characterization of circMagi1.** Sequence alignment of mmu_circ_0001496 and has_circ_0066459.

**Supplementary Fig. 2 | Construction of circMagi1 knockout mice. A.** (Left) Schematic representation of the circMagi1 knockout strategy, where intron deletion disrupts circMagi1 formation. (Right) PCR analysis using divergent and convergent primers to detect circMagi1 and linear Magi1 in cDNA and gDNA. Divergent primers amplified circMagi1 in WT mice cDNA but not in KO mice or gDNA. **B.** Pure heterozygote identification of the knockout mouse tail gene. **C-D.** Detection of circMagi1 (**C**) and linearMagi1 (**D**) gene expression in the lung tissues of circMagi1 knockout and wild-type mice. All data are presented as the mean ± SD. Statistical significance was assessed using unpaired t tests with Welch’s correction if necessary (C, D). ns, not signiﬁcant; ****P* < 0.001.

**Supplementary Fig. 3 | Cytokine expression in the lung tissues of circMagi1 knockout mice. A-E.** qPCR detection of Il-1β, Il-2, Tnf-α Ccl2 and Cxcl3 in mouse lungs. There were 5 mice in each group. All data are presented as the mean ± SD. Statistical significance was assessed using unpaired t tests with Welch’s correction if necessary (A-E). ns, not signiﬁcant; ***P* < 0.01; **P* < 0.05.

**Supplementary Fig. 4 | circMagi1 affects host immune function. A.** CyTOF analysis of T cells, B cells and NK cells within the CD45^+^ population. Each group included 3 mice, each mouse was infected with 4×10^6^ CFU, and whole lungs were collected for detection 24 hours after infection. **B.** Flow cytometry gating strategy for immune cells in lung tissues. **C.** Flow cytometry analysis of T cells, B cells and NK cells proportions in the wild-type mouse group and the circMagi1^-/-^ mouse group, with 8 mice in each group. Each mouse was infected with 3×10^6^ CFU, and whole lungs were collected for detection 24 hours after infection. All data are presented as the mean ± SD. Statistical significance was assessed using unpaired t tests with Welch’s correction if necessary (A,C). ns, not signiﬁcant; ****P* < 0.001; ***P* < 0.01; **P* < 0.05.

**Supplementary Fig. 5 | CircMagi1 binds to the G3BP2 protein and enhances its stability. A.** Validation of circMagi1–AGO2 interaction by RNA pulldown and RIP assays. circMagi1 and AGO2 were co-expressed in 293T cells for 48 h. Western blot analysis of pulldown and RIP samples confirmed AGO2 binding, and qPCR analysis detected circMagi1 enrichment in RIP samples, demonstrating a specific interaction between circMagi1 and AGO2. **B.** RIP assay verifying the interaction between circMagi1 and RPS6 in iBMDMs overexpressing circMagi1. Cell lysates were immunoprecipitated with an anti-RPS6 antibody. Western blot was performed on 10% of the RIP lysates to confirm successful pulldown of RPS6, and qPCR was performed on the remaining 90% to detect associated circMagi1. **C.** Validation of the efficiency and specificity of the biotin-labeled circMagi1 probe for RNA pulldown. 293T cells were transfected with circMagi1 for 48 hours, and cell lysates were incubated with either a circMagi1-specific or negative control (NC) probe to assess enrichment of circMagi1. **D.** Schematic diagram of the RNA pulldown. **E.** Schematic representation of truncated G3BP2 constructs and RIP validation of their interaction with circMagi1. **F.** qPCR detection of the effect of circMagi1 on G3BP2 levels. **G.** Western blot and quantification of G3BP2 in uninfected lung tissues from wild-type and circMagi1⁻/⁻ mice. **H.** qPCR detection of the effect of G3BP2 on circMagi1 levels in iBMDMs. All data are presented as the mean ± SD. Statistical significance was assessed using unpaired t tests with Welch’s correction if necessary (A, B, C, E, F, G, H). ns, not signiﬁcant; *****P* < 0.0001; ****P* < 0.001; **P* < 0.05.

**Supplementary Fig. 6 | Determination of G3BP2 RNA interference efficiency.** **A.** qPCR detection of G3BP2 expression in iBMDMs. **B.** Western blot analysis of G3BP2 expression in iBMDMs. **C.** Immumofluorescence detection of G3BP2 expression in iBMDMs. All data are presented as the mean ± SD. Statistical significance was assessed using unpaired t tests with Welch’s correction if necessary (A). *****P* < 0.0001.
