# Supplementary material for: Circular RNA circMagi1 regulates the host immune response in respiratory Pseudomonas aeruginosa infection through G3BP2
Source: mBio. 2026 Mar 23;17(4):e03617-25. doi: 10.1128/mbio.03617-25 (PMC13059810; doi:10.1128/mbio.03617-25)
Supplement: Supplemental Tables — Tables S1-S4. [file mbio.03617-25-s0008.docx]

**Supplementary Table 1. qPCR primers used in this study**

| **Primer** | **Sequences(5'-3')** | |
| --- | --- | --- |
| mmu_β-Actin | Forward primer | AGGGAAATCGTGCGTGACAT |
|  | Reverse primer | TCCAGGGAGGAAGAGGATGC |
| mmu_GAPDH | Forward primer | AGGTCGGTGTGAACGGATTTG |
|  | Reverse primer | TGTAGACCATGTAGTTGAGGTCA |
| hsa_β-Actin | Forward primer | CAATGAGCTGCGTGTGGCT |
|  | Reverse primer | GATAGCACAGCCTGGATAGCAA |
| has_circ_0066459 | Forward primer | ACCCCGAAGCGAACCAAGTCCT |
|  | Reverse primer | CGGGTTGTGCCTGTAAAGCTGC |
| mmu_circMagi1 | Forward primer | ACCCCTAAGCGAACAAAGTCCT |
|  | Reverse primer | CGGGTTGTGCCTGTAAAGCTAC |
| mmu_Magi1 | Forward primer | ACCCCTAAGCGAACAAAGTCCT |
|  | Reverse primer | CTGAGGGAACTTCTGAGAAGGG |
| mmu_G3BP2 | Forward primer | TGCCTCCTAGTGGTACTGTTT |
|  | Reverse primer | CTCGGCTGTGAGACTGGTG |
| mmu_IL-1β | Forward primer | AAATGCCACCTTTTGACAGTGATG |
|  | Reverse primer | GCTCTTGTTGATGTGCTGCTG |
| mmu_IL-2 | Forward primer | ATGAACTTGGACCTCTGCGG |
|  | Reverse primer | GTCCACCACAGTTGCTGACT |
| mmu_IL-10 | Forward primer | GGTTGCCAAGCCTTATCGGA |
|  | Reverse primer | AATCGATGACAGCGCCTCAG |
| mmu_TNF-α | Forward primer | GACGTGGAACTGGCAGAAGA |
|  | Reverse primer | ACTGATGAGAGGGAGGCCAT |
| mmu_CCL2 | Forward primer | GACCCCAAGAAGGAATGGGT |
|  | Reverse primer | ACAGAAGTGCTTGAGGTGGTT |
| mmu_CXCL3 | Forward primer | ACCCTACCAAGGGTTGATTTTGA |
|  | Reverse primer | TGACTTCTGTCTGGGTGCAGT |

**Supplementary Table 2. Patient Alveolar Lavage Subgroup Information**

|  | **Patient** | **Sex/age** | **Symptoms** | **chest computed tomography** | **Pathogen culture** | | **Antigens** |
| --- | --- | --- | --- | --- | --- | --- | --- |
|  |  |  |  |  | **sputum** | **Lavage** |  |
| Control | H.L | F/54 | Pulmonary space occupying lesion | Multiple cavities in the right lung with soft tissue masses | - | + | - |
|  | X.G | F/43 | Pulmonary space occupying lesion(Nodule in the upper lobe of the right lung) | ND | - | - | ND |
|  | X.P | F/41 | Pulmonary space occupying lesion | Right upper lung cavity | - | - | ND |
|  | X.Y | M/57 | Pulmonary space occupying lesion(right middle lobe occupancy) | ND | - | - | ND |
|  | P.L | F/56 | Bilateral pneumonic nodules | ND | - | - | ND |
|  | L.Y | M/50 | Chronic bronchitis | mass shadow of right lower lung fragment | - | + | - |
|  | Q.X | F/47 | pulmonary alveolar proteinosis | ND | + | + | - |
|  | Y.C | F/40 | Pulmonary space occupying lesion | Ground-glass nodular shadows in the right upper lung, scattered small nodular shadows in both lungs | - | - | ND |
|  | Z.S | F/56 | Pulmonary space occupying lesion(right middle lobe) | Ground-glass nodular shadows in the middle lobe of the right lung and the upper lingual branch of the left lung | - | - | ND |
|  | S.H | M/60 | Bronchiectasis | Right middle lobe and left lower lobe branching enlargement | + | + | - |
| Infection | F.H | M/57 | Bronchiectasis with Pseudomonas aeruginosa infection | ND | - | - | ND |
|  | S.L | F/70 | Bronchiectasis with infection | Multiple bronchiectasis in both lower lungs, multiple nodules with calcified foci in both lungs | + | + | *P. aeruginosa、C. albicans* |
|  | H.Z | M/68 | Bronchiectasis with infection | Branchial spread with infection in both lungs | + | + | *P. aeruginosa、X. achromobacteria* |
|  | T.H | M/67 | pulmonary infection | Soft tissue shadows in the upper lobe of the right lung, solid lesions, atelectasis, and branched expansion in the middle and lower lobes of the right lung | - | + | ND |
|  | F.L | F/66 | Bronchiectasis with infection | Scattered branching in both lungs | + | - | *P. aeruginosa* |
|  | C.W | F/61 | Bronchiectasis with infection | Scattered bronchiectasis in both lungs, more prominent in the upper lobe of the right lung | + | + | *P. aeruginosa* |
|  | C.D | F/64 | Bronchiectasis with infection | Branchial expansion with mucus plugs in both lungs, multiple patchy, nodular shadows in both lungs | + | + | *C. albicans* |
|  | X.H | M/59 | Bronchodilatation of both lungs with infection | Multiple branched expansions in both lungs | - | + | *P. aeruginosa、A. niger* |
|  | X.W | F/51 | Bronchodilatation of both lungs with infection | Scattered branching in both lungs | - | + | *P. aeruginosa、K. pnenmoniae* |
|  | X.C | F/38 | Malignant tumor in left lung | ND | + | - | *C. glabrata* |

**Supplementary Table 3. Detailed gene list for enriched pathways for Figure 2B**

| **KEGG ID** | **Description** | **geneName** | **Count** |
| --- | --- | --- | --- |
| mmu04060 | Cytokine-cytokine receptor interaction | Csf1/Csf2rb2/Csf2rb/Gdf15/Cxcl2/Tnf/Osm/Il1rn/Lif/Csf3/Il1a/Cd40/Tnfrsf12a/Amhr2/Ccl5/Il1b/Il17rc/Pf4/Il18/Cxcr3/Cxcl3/Ccr1/Tnfsf10 | 23 |
| mmu04940 | Type I diabetes mellitus | Cd28/Tnf/H2-M2/Il1a/Ptprn/Il1b/H2-M3/Gm9655/H2-Q4 | 9 |
| mmu05332 | Graft-versus-host disease | Cd28/Tnf/H2-M2/Il1a/Il1b/H2-M3/H2-Q4 | 7 |
| mmu04668 | TNF signaling pathway | Csf1/Cxcl2/Tnf/Lif/Jag1/Edn1/Ccl5/Il1b/Ifi47/Ptgs2/Irf1/Cxcl3/Pik3r3/Vcam1 | 14 |
| mmu05330 | Allograft rejection | Cd28/Tnf/H2-M2/Cd40/H2-M3/H2-Q4 | 6 |
| mmu04621 | NOD-like receptor signaling pathway | Gbp2/Gbp3/Cxcl2/Tnf/Gbp7/Ifi204/Gbp5/Irf7/Oas3/Ccl5/Il1b/Irgm2/Il18/Cxcl3/Map1lc3a/Gm18445 | 16 |
| mmu04064 | NF-kappa B signaling pathway | Malt1/Cxcl2/Tnf/Ddx58/Cd40/Bcl2a1d/Il1b/Ptgs2/Cxcl3/Gadd45g/Vcam1 | 11 |
| mmu04512 | ECM-receptor interaction | Spp1/Itgb4/Itgb3/Agrn/Lamc2/Col4a2/Thbs3/Hspg2 | 8 |
| mmu04620 | Toll-like receptor signaling pathway | Ctsk/Spp1/Tnf/Irf7/Cd40/Ccl5/Il1b/Tlr3/Pik3r3 | 9 |
| mmu04640 | Hematopoietic cell lineage | Csf1/Tnf/Anpep/Itgb3/Csf3/Il1a/Cd33/Il1b | 8 |

**Supplementary Table 4. Antibodies used for CyTOF staining**

| **Mag-Tag** | **Antibodies** | **Clone** | **Stain** | **Vendor** | **Catalot** |
| --- | --- | --- | --- | --- | --- |
| 89Y | CD45 | 30-F11 | Surface | BioLegend | 103102 |
| 115In | CD3ε | 145-2C11 | Surface | BioLegend | 100302 |
| 139La | Ki-67 | SolA15 | Surface | eBioscience | 14-5698-82 |
| 141Pr | CD103 | 2E7 | Surface | Biolegend | 121402 |
| 142Nd | MHC II(I-A/I-E) | M5/114.15.2 | Surface | BioLegend | 107602 |
| 143Nd | KLRG1 | 2F1 | Surface | eBioscience | 16-5893-82 |
| 144Nd | CX3CR1 | SA011F11 | Surface | BioLegend | 149002 |
| 145Nd | CD161(NK1.1) | PK136 | Surface | BioLegend | 108702 |
| 146Nd | Ly-6C | HK1.4 | Surface | BioLegend | 128002 |
| 147Sm | Ly-6G | 1A8 | Surface | BioLegend | 127602 |
| 148Nd | CD197(CCR7) | 4B12 | Surface | BioLegend | 120101 |
| 149Sm | CD19 | 6D5 | Surface | BioLegend | 115502 |
| 150Nd | CD127(IL-7Rα) | A7R34 | Surface | BioLegend | 135002 |
| 151Eu | CD44 | IM7 | Surface | BioLegend | 103002 |
| 152Sm | CD5 | 53-7.3 | Surface | BioLegend | 100602 |
| 153Eu | TCR γ/δ | GL3 | Surface | BioLegend | 118101 |
| 154Sm | CD11c | N418 | Surface | BioLegend | 117302 |
| 155Gd | CD317(BST-2,Tetherin) | 44E9R | Surface | R&D Systems | MAB8660 |
| 156Gd | CD68 | FA-11 | Surface | BioLegend | 137002 |
| 157Gd | CD335(NKp46) | 29A1.4 | Surface | BioLegend | 137602 |
| 158Gd | IgM | RMM-1 | Surface | BioLegend | 406502 |
| 159Tb | F4/80 | Cl:A3-1 | Surface | Bio-Rad | MCA497G |
| 160Gd | TCR β chain | H57-597 | Surface | BioLegend | 109202 |
| 161Dy | CD200(OX2) | OX-90 | Surface | BioLegend | 123802 |
| 162Dy | CD25(IL-2Rα) | 3C7 | Surface | BioLegend | 101902 |
| 163Dy | CD27 | LG.3A10 | Surface | Biolegend | 124202 |
| 164Dy | RORγ(RORC) | 600214 | Surface | R&D Systems | MAB6109 |
| 165Ho | T-bet | 4B10 | Intracellular | BioLegend | 644802 |
| 166Er | CD314(NKG2D) | CX5 | Surface | BioLegend | 130202 |
| 167Er | CD206(MMR) | C068C2 | Intracellular | Biolegend | 141702 |
| 168Er | Foxp3 | FJK-16s | Intracellular | eBioscience | 14-5773-82 |
| 169Tm | CD62L | MEL-14 | Surface | BioLegend | 104402 |
| 170Er | Ly-6A/E(Sca-1) | D7 | Surface | BioLegend | 108135 |
| 171Yb | GATA-3 | TWAJ | Surface | eBioscience | 14-9966-82 |
| 172Yb | CD279(PD-1) | 29F.1A12 | Surface | BioLegend | 135202 |
| 173Yb | CD172a(SIRPα) | P84 | Surface | BioLegend | 144002 |
| 174Yb | CD192(CCR2) | 475301 | Surface | R&D Systems | MAB55381-100 |
| 175Lu | Siglec-F | E50-2440 | Surface | BD Biosciences | 552125 |
| 176Yb | MERTK(Mer) | 2B10C42 | Surface | BioLegend | 151502 |
| 197Au | CD4 | RM4-5 | Surface | BioLegend | 100520 |
| 198Pt | CD8a | 53-6.7 | Surface | BioLegend | 100746 |
| 209Bi | CD11b | M1/70 | Surface | BioLegend | 117302 |
